# A Decision Aid to Support Tubal Sterilization Decision-Making Among Pregnant Women: The MyDecision/MiDecisión Randomized Clinical Trial

**DOI:** 10.1001/jamanetworkopen.2024.2215

**Published:** 2024-03-19

**Authors:** Sonya Borrero, Elizabeth A. Mosley, Michaella Wu, Christine Dehlendorf, Catherine Wright, Kaleab Z. Abebe, Nikki Zite

**Affiliations:** 1 Division of General Internal Medicine, Department of Medicine, University of Pittsburgh, Pittsburgh, Pennsylvania; 2 Center for Innovative Research on Gender Health Equity, University of Pittsburgh, Pittsburgh, Pennsylvania; 3 Department of Family & Community Medicine, University of California, San Francisco; 4 Center for Research on Health Care Data Center, University of Pittsburgh, Pittsburgh, Pennsylvania; 5 Department of Obstetrics and Gynecology, University of Tennessee Graduate School of Medicine, Knoxville

## Abstract

**Importance:**

Tubal sterilization is common, especially among individuals with low income. There is substantial misunderstanding about sterilization among those who have undergone the procedure, suggesting suboptimal decision-making about a method that permanently ends reproductive capacity.

**Objective:**

To test the efficacy of a web-based decision aid for improving tubal sterilization decision quality.

**Design, Setting, and Participants:**

This randomized clinical trial conducted between March 2020 and November 2023 included English- or Spanish-speaking pregnant cisgender women aged 21 to 45 years who had Medicaid insurance and were contemplating tubal sterilization after delivery. Participants were recruited from outpatient obstetric clinics in 3 US cities.

**Intervention:**

Participants were randomized 1:1 to usual care (control arm) or to usual care plus a web-based decision aid (MyDecision/MiDecisión) (intervention arm). The aid includes written, audio, and video information about tubal sterilization procedures; an interactive table comparing contraceptive options; values-clarifying exercises; knowledge checks; and a summary report.

**Main Outcomes and Measures:**

The co–primary outcomes were tubal sterilization knowledge and decisional conflict regarding the contraceptive decision. Knowledge was measured as the percentage of correct responses to 10 true-false items. Decisional conflict was measured using the low-literacy Decision Conflict Scale, with lower scores on a range from 0 to 100 indicating less conflict.

**Results:**

Among the 350 participants, mean (SD) age was 29.7 (5.1) years. Compared with the usual care group, participants randomized to the decision aid had significantly higher tubal sterilization knowledge (mean [SD] proportion of questions answered correctly, 76.5% [16.9%] vs 55.6% [22.6%]; *P* < .001) and lower decisional conflict scores (mean [SD], 12.7 [16.6] vs 18.7 [20.8] points; *P* = .002). The greatest knowledge differences between the 2 groups were for items about permanence, with more participants in the intervention arm answering correctly that tubal sterilization is not easily reversible (90.1% vs 39.3%; odds ratio [OR], 14.2 [95% CI, 7.9-25.4]; *P* < .001) and that the tubes do not spontaneously “come untied” (86.6% vs 33.7%; OR, 13.0 [95% CI, 7.6-22.4]; *P* < .001).

**Conclusions and Relevance:**

MyDecision/MiDecisión significantly improved tubal sterilization decision-making quality compared with usual care only. This scalable decision aid can be implemented into clinical practice to supplement practitioner counseling. These results are particularly important given the recent increase in demand for permanent contraception after the US Supreme Court decision overturning federal abortion protections.

**Trial Registration:**

ClinicalTrials.gov Identifier: NCT04097717

## Key Points

**Question:**

Can a web-based decision aid improve sterilization decision quality among pregnant individuals with Medicaid insurance who are considering permanent contraception?

**Findings:**

In this multisite randomized clinical trial of 350 pregnant participants, those assigned to use the MyDecision/MiDecisión tool supporting informed and value-concordant decision-making had significantly greater tubal sterilization knowledge and lower decisional conflict compared with those assigned to usual care.

**Meaning:**

MyDecision/MiDecisión may help patients with Medicaid insurance make informed and value-concordant decisions about a contraceptive method that permanently ends reproductive capacity and for which there is a history of coercive practice.

## Teaser

This randomized clinical trial examines the efficacy of a web-based decision aid to increase knowledge about tubal sterilization and reduce decisional conflict among pregnant women considering permanent contraception.

## Introduction

Tubal sterilization is the most commonly used contraceptive method among US women aged 15 to 49 years,^1^ and preliminary data indicate that the demand for permanent contraception is increasing in the context of diminishing abortion access following the June 2022 US Supreme Court *Dobbs v Jackson Women’s Health Organization* decision.^2,3^ Tubal sterilization is disproportionately used by people with social disadvantages, including those with low income, with lower educational level, from marginalized racial and ethnic groups, and with public insurance.^4,5,6^ As these same groups have been targeted by coercive sterilization practices and are more likely to experience poststerilization regret,^7,8,9,10^ there is a critical need to ensure fully informed and supported decision-making about this procedure that permanently ends reproductive capacity.

Research exploring decision-making about permanent contraception, however, has revealed that people’s preferences and subsequent decisions are not always well informed. In 1 study^11^ among women who had undergone tubal sterilization, nearly half incorrectly believed that a reversal procedure could easily restore fertility, over a third incorrectly believed that the fallopian tubes would grow back together or unblock themselves after a few years, and a quarter did not know there are reversible methods of contraception as effective as bilateral tubal ligation for pregnancy prevention. These findings are consistent with other research documenting widespread misinformation^12,13,14^ and suggest suboptimal decision-making and inadequate practitioner counseling for a high-stakes procedure.

Interventions are needed to support practitioner counseling and facilitate optimal decision-making about permanent contraception, particularly for communities whose reproduction has been devalued by society. Our team developed a patient-facing decision aid (MyDecision/MiDecisión) to support informed and value-concordant decision-making among individuals with low income considering tubal sterilization.^15^ A decision support tool is particularly useful in this context because sterilization is a preference-sensitive decision with multiple contraceptive alternatives and known high levels of misunderstanding about key aspects of the method, including its permanent nature. This study sought to test the efficacy of MyDecision/MiDecisión for sterilization decision quality in a multisite randomized clinical trial (RCT). Specifically, we hypothesized that study participants randomized to receive the decision aid would have greater knowledge about sterilization and alternative contraceptive options and lower decisional conflict compared with those randomized to usual care.

## Methods

### Study Design

We conducted a multisite, parallel-arm RCT (NCT04097717) between March 2020 and November 2023 among pregnant women to evaluate the effect of MyDecision/MiDecisión compared with usual prenatal care on participants’ postpartum contraceptive decision-making. The trial protocol (Supplement 1) was approved at each trial site and was overseen by the University of Pittsburgh institutional review board. Verbal or written consent was obtained from participants. This study followed the Consolidated Standards of Reporting Trials (CONSORT) reporting guideline.^16^

### Study Participants and Recruitment

Participants were recruited from outpatient obstetric clinics in Pittsburgh, Pennsylvania; Knoxville, Tennessee; and San Francisco, California. Using electronic medical records (EMRs), study staff identified pregnant people with Medicaid insurance who were scheduled for a first- or second-trimester prenatal appointment because data indicate that people contemplating postpartum permanent contraception often do so soon after the news of their pregnancy.^17^ Given varying regulatory and COVID-19–related procedures, depending on the participating site, patients were either sent letters, approached by practitioners or study staff in the clinic, or called directly to initiate discussions about interest in the study. In addition to EMR-based recruitment, study fliers were posted at clinic sites and online (eg, on a classified advertisement website). Interested participants’ preferences for in-person or remote participation were accommodated to complete screening, consent, baseline assessment, the intervention, and the initial (time 1 [T1]) assessment, ideally in a single encounter.

Race and ethnicity were ascertained by self-report and included in the analysis to ensure a racially diverse sample and test for potentially differential effects of the decision aid across racial and ethnic groups given the history of coercive sterilization practices targeting marginalized racial and ethnic populations. Categories included Hispanic, Latina, or of Spanish origin (hereafter, Hispanic); non-Hispanic Black (hereafter, Black); non-Hispanic White (hereafter, White); non-Hispanic multiracial (hereafter, multiracial); and non-Hispanic other (hereafter, other; American Indian, Asian, Native Alaskan, Native Hawaiian, or Pacific Islander). As the Hispanic populations in Pennsylvania and Tennessee are relatively small, we relied on recruitment efforts in San Francisco to ensure Hispanic representation in the study sample. Thus, we had bilingual staff at that site, and participants in San Francisco were able to complete study assessments in either English or Spanish; participants at all other sites completed study assessments in English. However, all participants were able to use the decision aid in either English or Spanish according to their preference. Eligibility requirements included (1) being less than 24 weeks pregnant (to capture people early enough in pregnancy to be deliberating their postpartum contraception), (2) considering tubal sterilization after delivery, (3) being enrolled in or eligible for Medicaid insurance (to focus on individuals with low income, who have historically experienced sterilization abuses), (4) being fluent in English or Spanish, and (5) being aged 21 to 45 years (to comply with federal requirements that prohibit federally funded sterilization for people aged younger than 21 years).

### Study Procedures

All study participants completed baseline assessments and were randomized to either the intervention arm or the usual care arm in a 1:1 allocation ratio using permuted block randomization that was integrated into the web-based data collection system and stratified by site. Neither the researchers nor the participants were blinded to the intervention assignment. Participants randomized to the intervention arm were asked to complete the web-based decision aid immediately after randomization on a personal or university electronic device. Research assistants helped participants log into the decision aid platform with a unique user identification and password. Participants then used the decision aid on their own. The T1 assessments, including ascertainment of our primary outcomes, were completed immediately after going through the decision aid (intervention arm) or baseline assessment (control arm), which occurred prior to 24 weeks’ gestation. Subsequent assessments were conducted via telephone during the third trimester (T2) and at 3 months postpartum. Consent, randomization, and all data collection were conducted using the REDCap platform hosted at the University of Pittsburgh.

### Study Arms

Participants randomized to the intervention arm used the MyDecision/MiDecisión tool. Details about the development and content of the decision aid have been reported elsewhere.^15^ In brief, the decision aid was produced using a systematic approach in accordance with best practices for decision aid development and with guidance from a multidisciplinary steering committee comprising reproductive justice advocates, practitioners, social and decision scientists, an ethicist, and people with lived sterilization decision-making experience. The aid includes written, audio, and video information about tubal sterilization procedures; an interactive table comparing contraceptive options; values-clarifying exercises; knowledge checks; and a summary report that participants could share with their practitioner. As practitioners were free to counsel patients as they normally would, the intervention was the decision aid plus usual care. The control was usual care only.

### Outcomes

Consistent with best practices for evaluating the efficacy of decision aids,^18^ we assessed the following co–primary outcomes to determine decision quality: (1) tubal sterilization knowledge and (2) participant decisional conflict. Tubal sterilization knowledge was measured as the percentage of correct responses to 10 previously published true-false items about key aspects of tubal sterilization and alternative contraceptive options.^11^ Participant decisional conflict was measured using the low-literacy version of the validated Decision Conflict Scale, which assesses 4 dimensions of decision-making—feeling informed, value clarity, support, and feeling uncertain—using Likert-scale items.^19^ The overall Decision Conflict Scale score ranges from 0 to 100, with lower scores indicating lower conflict or greater certainty.

### Sample Size

We based the sample size on the standardized mean differences for the co–primary outcomes of knowledge (Cohen *d*, 0.46) and decisional conflict (Cohen *d*, 0.36) reported in the 2014 Cochrane review of decision aid RCTs.^20^ Assuming a 2-sided α of .025 for each co–primary outcome and 15% attrition, 175 women per arm would afford us 80% power to detect these differences.^21^

### Statistical Analysis

Both primary outcomes were analyzed as continuous variables (score range of 0-100) using linear regression as a function of study arm and study site. Co–primary outcomes are reported as mean differences with 97.5% CIs. Differences between the intervention and control groups on individual knowledge questions (correct vs incorrect) were tested using multivariable logistic regression controlling for study site, and differences on decisional conflict subscales were tested using multivariable linear regression controlling for study site. We used an intention-to-treat analysis in which all participants’ data were analyzed according to their intervention assignment. No outcome data were excluded.

We conducted subgroup analyses to identify any difference in intervention efficacy based on the following: race and ethnicity, age, language of the consent form, mode of decision aid completion (in person or virtual), recruitment site, educational level, and whether the participant had received any practitioner counseling about permanent contraception during the current pregnancy. These analyses were accomplished by constructing linear regression models in which the study arm, the study site, the subgroup variable, and an interaction term between that subgroup variable and the study arm were included as independent variables. Finally, as an exploratory analysis, we reassessed knowledge and decisional conflict at T2 to determine the durability of the intervention into the third trimester. Each co–primary hypothesis was tested at the .025 α level; analyses for all other hypotheses were tested at the .05 α level. All analyses were conducted using Stata, version 18 (StataCorp LLC).

## Results

### Participant Characteristics

**Figure 1.**
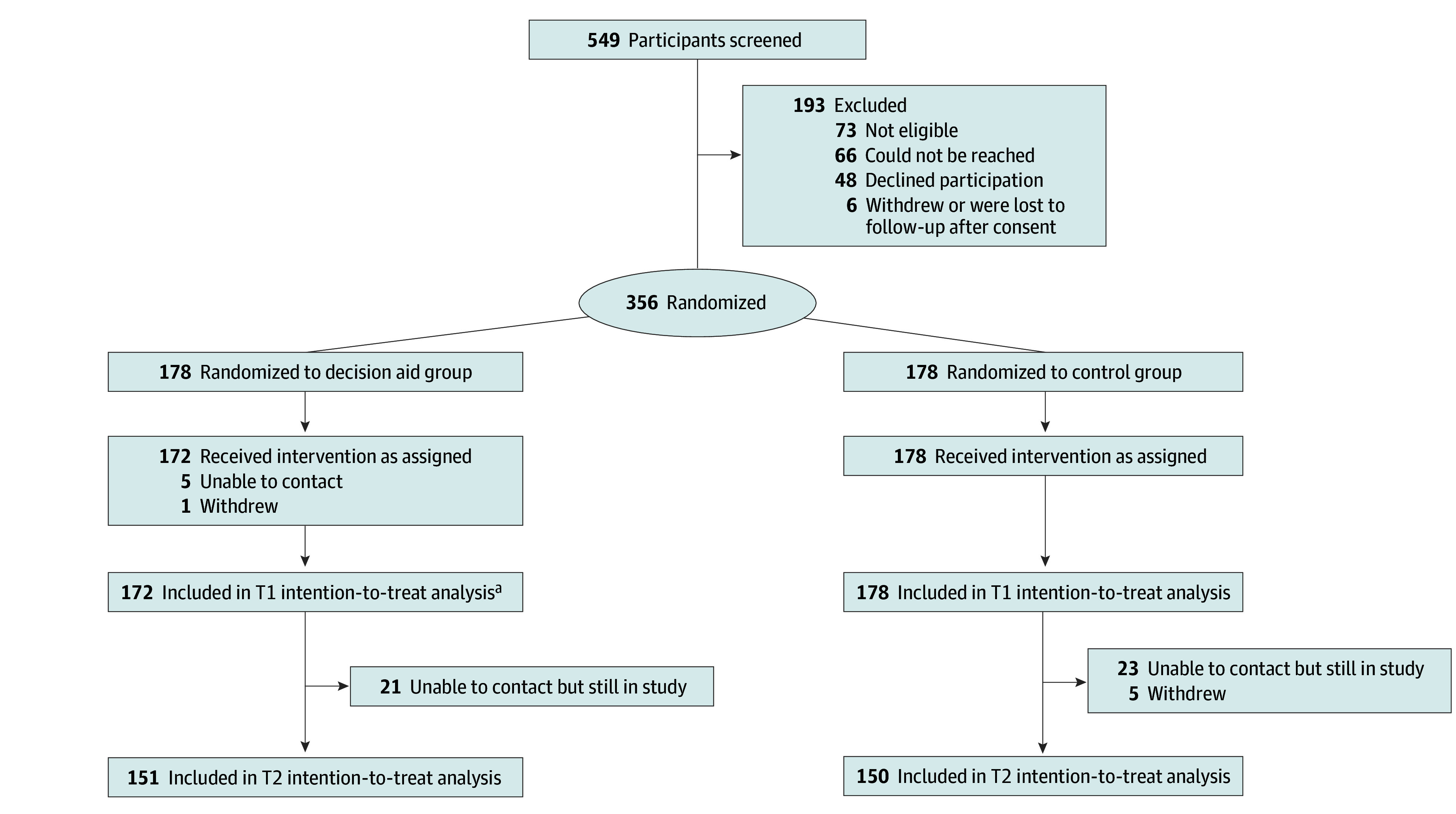
CONSORT Diagram of Recruitment and Data Collection Withdrawal was defined as anyone who withdrew from the study voluntarily. T1 indicates time 1, at the completion of the initial assessment; T2, time 2, during the third trimester. ^a^Two participants did not open the decision aid but completed the assessment and were included in the intention-to-treat analysis.

A total of 356 cisgender women were randomized, 178 to each group. In the intervention group, 5 were unable to be contacted for assessment, and 1 withdrew; in the control arm, no individuals were lost to follow-up, thus leaving 350 participants for primary outcome analyses at T1 (Figure 1). Mean (SD) age of participants was 29.7 (5.1) years, and mean gestational age was 16.1 weeks (IQR, 12.7-20.1 weeks) (Table 1; eTable 1 in Supplement 2 shows site-specific demographics). Overall, 91 participants (26.0%) identified as Black, 91 (26.0%) as Hispanic, 137 (39.1%) as White, 14 (4.0%) as multiracial, and 14 (4.0%) as other race and ethnicity. The mean amount of time spent using the decision aid was 14.36 minutes in English and 18.45 minutes in Spanish.

**Table 1.** Participant Demographics and Baseline Characteristics

Variable	Participants^a^		
	All (N = 350)	Intervention arm (n = 172)	Control arm (n = 178)
Age, mean (SD), y	29.7 (5.1)	29.4 (4.7)	30.1 (5.4)
Self-identified race and ethnicity			
Hispanic, Latina, or Spanish origin	91 (26.0)	46 (26.7)	45 (25.3)
Non-Hispanic Black	91 (26.0)	46 (26.7)	45 (25.3)
Non-Hispanic White	137 (39.1)	63 (36.6)	74 (41.6)
Non-Hispanic multiracial	14 (4.0)	8 (4.7)	6 (3.4)
Non-Hispanic other^b^	14 (4.0)	8 (4.7)	6 (3.4)
Educational level			
<High school	41 (11.7)	22 (12.8)	19 (10.7)
High school or GED	165 (47.1)	82 (47.7)	83 (46.6)
Some college or more	143 (40.9)	68 (39.5)	75 (42.1)
Relationship status			
Single or never married	95 (27.1)	48 (27.9)	47 (26.4)
Married or cohabiting	224 (64.0)	109 (63.4)	115 (64.6)
Previously married	13 (3.7)	5 (2.9)	8 (4.5)
Other or missing	18 (5.1)	10 (5.8)	8 (4.5)
Percentage of federal poverty level			
<100	197 (56.3)	98 (57.0)	99 (55.6)
100-199	134 (38.3)	64 (37.2)	70 (39.3)
>200	10 (2.9)	5 (2.9)	5 (2.8)
Missing	9 (2.6)	5 (2.9)	4 (2.3)
Poor or marginal health literacy^c^	59 (16.9)	28 (16.3)	31 (17.4)
Children excluding current pregnancy, mean (SD), No.	2.16 (1.4)	2.1 (1.3)	2.3 (1.6)
Gestational age, mean (IQR), y	16.1 (12.7-20.1)	16.3 (12.8-20.2)	15.9 (12.6-19.6)
Reported receiving any contraceptive counseling during this pregnancy	185 (52.9)	94 (54.7)	91 (51.1)
Reported receiving sterilization counseling during this pregnancy	162 (46.3)	84 (48.8)	78 (43.8)
Reported signing Medicaid consent form	8 (2.3)	6 (3.5)	2 (1.1)

Abbreviation: GED, General Educational Development.

^a^ Data are presented as the number (percentage) of participants unless otherwise indicated.

^b^ Other included American Indian, Asian, Native Alaskan, Native Hawaiian, and Pacific Islander.

^c^ Defined by patients responding to a 5-point, single-item literacy screening question: “How confident are you filling out medical forms by yourself?” Answers included “somewhat confident,” “a little bit confident,” or “not at all confident.”

### Primary Outcomes

**Figure 2.**
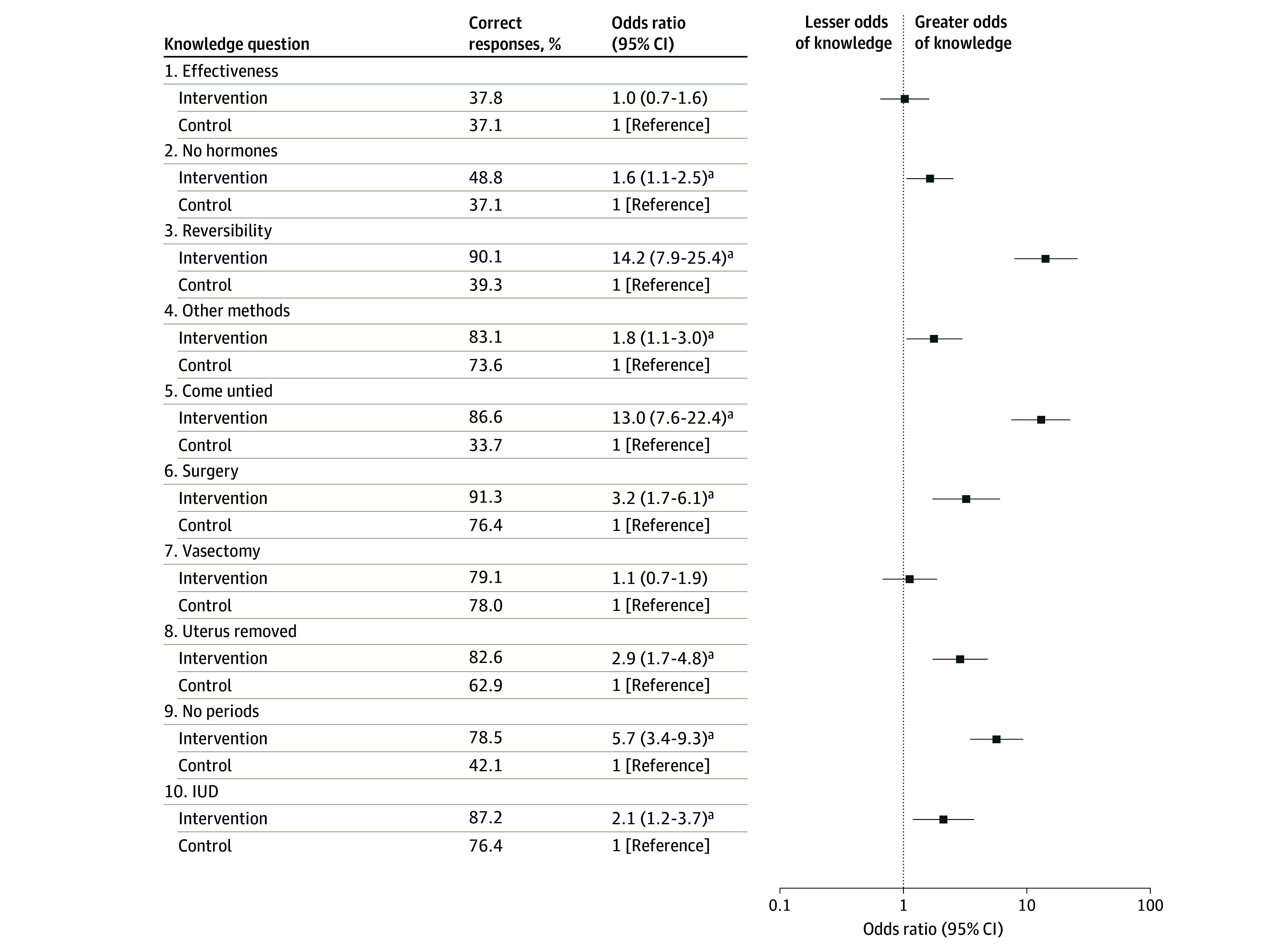
Percentage of Correct Responses to Tubal Sterilization Knowledge Questions by Study Arm Odds ratios were calculated using multivariate logistic regression and were adjusted for recruitment site. Possible responses to questions were “true” (T), “false” (F), or “don’t know.” Questions included the following (correct answers in parentheses): (1) tubal sterilization is 100% effective at preventing pregnancy (F); (2) tubal sterilization is the only method of birth control that does not contain hormones (F); (3) after a woman has had her tubes tied, her doctor can easily reverse the procedure if she wants to get pregnant (F); (4) there are other methods of birth control that are as effective as tubal sterilization for preventing pregnancy, but can be stopped or removed if a woman decides that she wants to get pregnant (T); (5) after getting a tubal sterilization, the tubes are likely to “come untied,” grow back together, or unblock on their own (F); (6) tubal sterilization is a surgery that requires some form of anesthesia (T); (7) after a man has a vasectomy, he will still be able to ejaculate when he has an orgasm (or “comes” during intercourse) (T); (8) a woman’s uterus and ovaries are removed during a tubal sterilization procedure (F); (9) a woman should expect to have no or lighter periods after getting her tubes tied (F); and (10) once an intrauterine device (IUD) or contraceptive implant is inserted in the body, it can prevent pregnancy for several years (T). ^a^*P* < .05.

**Figure 3.**
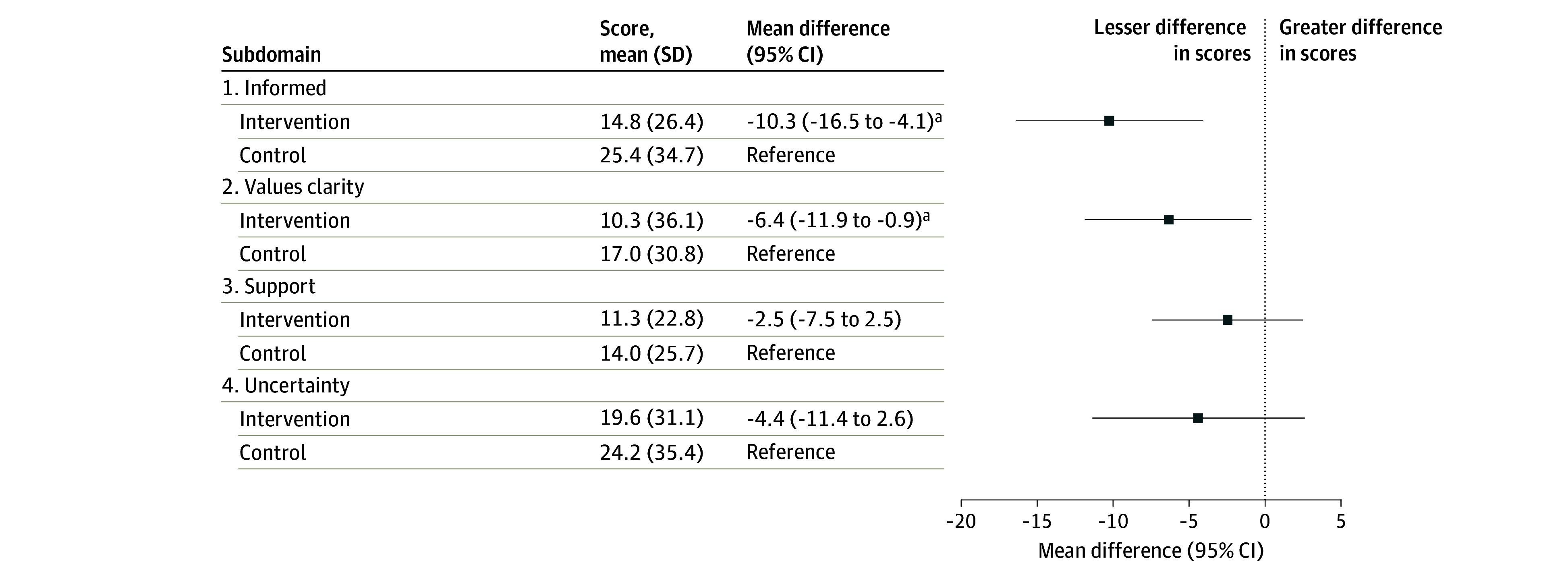
Mean Scores for 4 Subdomains of Decisional Conflict by Study Arm Score range was 0 to 100, with higher scores representing greater conflict. Mean scores were calculated after adjusting for recruitment site, and mean differences were calculated using multivariate linear regression. Possible responses were “yes,” “no,” or “unsure.” Questions in the “informed” subdomain included (1) Do you know which options are available to you? (2) Do you know the benefits of each option? and (3) Do you know the risks and side effects of each option? Questions in the “values clarity” subdomain included (4) Are you clear about which benefits matter most to you? and (5) Are you clear about which risks and side effects matter most to you? Questions in the “support” subdomain included (6) Do you have enough support from others to make a choice? (7) Are you choosing without pressure from others? and (8) Do you have enough advice to make a choice? Questions in the “uncertainty” subdomain included (9) Are you clear about the best choice for you? and (10) Do you feel sure about what to choose? Intervention group, n = 172; control group, n = 178. ^a^Informed domain, *P* < .001; values clarity domain, *P* = .02.

Tubal sterilization knowledge was significantly higher among those in the decision aid group compared with those in the control group (mean [SD] percentage of 10 items answered correctly, 76.5% [16.9%] vs 55.6% [22.6%]; mean difference, 20.8 percentage points [97.5% CI, 16.2-25.4 percentage points]; Cohen *d*, 1.05 [97.5% CI, 0.79-1.31]; *P* < .001) (Table 2). Results for each true-false question are summarized in Figure 2. The largest differences in knowledge scores between the study groups were observed for the 2 items about the permanence of the procedure: compared with those in the control arm, more participants randomized to the decision aid answered correctly that tubal sterilization is not easily reversible (90.1% vs 39.3%; odds ratio [OR], 14.2 [95% CI, 7.9-25.4]; *P* < .001) and that the tubes do not spontaneously “come untied” (86.6% vs 33.7%; OR, 13.0 [95% CI, 7.6-22.4]; *P* < .001). Those randomized to the decision aid experienced significantly lower decisional conflict compared with those receiving usual care alone (mean [SD] score, 12.7 [16.6] vs 18.7 [20.8] points; mean difference, −5.8 percentage points [97.5% CI: −10.0 to −1.6 percentage points]; Cohen *d*, −0.31 [97.5% CI, −0.55 to −0.07]; *P* = .002) (Table 2). The biggest differences were observed for the informed subscale (mean [SD] score, 14.8 [26.4] vs 25.4 [34.7]; *P* = .001) and the values subscale (mean [SD] score, 10.3 [26.1] vs 17.0 [30.8] points; *P* = .02) (Figure 3).

**Table 2.** Primary Outcomes for Tubal Sterilization Knowledge and Decisional Conflict From the MyDecision/MiDecisión Randomized Clinical Trial

Outcome	Mean (SD)		Mean difference (97.5% CI)^a^	Cohen *d* (97.5% CI)	*P* value
	Decision aid	Control			
Knowledge about tubal sterilization, %^^b^^	76.5 (16.9)	55.6 (22.6)	20.8 (16.2-25.4)	1.05 (0.79 to 1.31)	<.001
Decisional conflict in postpartum contraceptive choice, score^^c^^	12.7 (16.6)	18.7 (20.8)	−5.8 (−10.0 to −1.6)	−0.31 (−0.55 to −0.07)	.002

^a^ Adjusted for study site.

^b^ Based on a scale of 0% to 100%, calculated from the percentage of the 10 questions answered correctly.

^c^ Based on a scale of 0 to 100 points, where 0 is the least decisional conflict and 100 is the most decisional conflict.

### Exploratory Analyses

The improvements in knowledge and decisional conflict at T1 did not significantly differ by race and ethnicity, language, age, mode of decision aid completion, study site, educational level, or whether the participant reported practitioner sterilization counseling during the current pregnancy (eTable 2 in Supplement 2). However, the reduction in decisional conflict was greater for people who consented in Spanish compared with those who consented in English (mean difference, −18.2 points [95% CI, −31.6 to −4.9 points] vs −3.7 points [95% CI, −7.0 to −0.3 points]; *P* = .003).

By T2 (third trimester; mean gestational duration, 32.8 weeks [IQR, 32.0-34.0 weeks]), most participants in both the intervention group (140 of 164 [85.4%]) and the control group (134 of 156 [85.9%]) reported having received practitioner counseling about contraception and/or sterilization. Tubal sterilization knowledge among the intervention group was still a mean of 6.7 percentage points (95% CI, 2.2-11.1 percentage points) higher than in the control group (mean [SD] proportion of correct answers, 67.9% [19.9%] vs 61.4% [20.8%]; Cohen *d*, 0.33 [97.5% CI, 0.12-0.54]; *P* = .003). Notably, the 2 questions about permanence still had the largest knowledge difference between the intervention and control groups, with participants randomized to the decision aid more likely to answer correctly that tubal sterilization is not easily reversible (76.0% vs 54.4%; OR, 2.7 [95% CI, 1.6-4.4]; *P* < .001) and that the tubes do not spontaneously “come untied” (69.5% vs 40.9%; OR, 3.3 [95% CI, 2.1-5.4]; *P* < .001). Decisional conflict scores were a mean of 4.1 points (95% CI, 7.8-0.4 points) lower for decision aid participants compared with the control group (mean [SD] score, 9.6 [15.8] points vs 13.5 [19.5] points; Cohen *d*, −0.23 [97.5% CI, −0.44 to −0.02]; *P* = .03). Only the informed subscale had significant differences between the intervention and control groups (mean [SD] score, 9.5 [22.5] points vs 16.1 [27.9] points; *P* = .02).

## Discussion

In this RCT testing the efficacy of a web-based decision aid, MyDecision/MiDecisión, on tubal sterilization decision-making in a sample of pregnant people enrolled in Medicaid, we found that compared with usual care only, the decision aid significantly increased knowledge by a mean of 20.8 percentage points and decreased decisional conflict by a mean of 5.8 points. This translates to effect sizes (Cohen *d*) of 1.05 and −0.31, respectively. Given that Cohen *d* can be interpreted as large (0.8), medium (0.5), and small (0.2) effect sizes,^22^ we concluded that the decision aid was associated with large improvements in knowledge and small to medium improvements in decisional conflict. Importantly, the improved knowledge and decisional conflict after use of the decision aid were observed across all age groups, racial and ethnic groups, educational levels, and study sites and for those who had or had not already received some practitioner counseling about permanent sterilization. Notably, the improvement in decisional conflict was greater for Spanish-speaking individuals than for English-speaking individuals. These findings have important clinical and policy implications.

Findings from our study underscore the potential of a decision aid to address the shortcomings of practitioner counseling by offering an independent, patient-directed path to obtain meaningful information and to clarify one’s own values to reduce decisional conflict. While informed and value-concordant decision-making is important for any contraceptive method, it is especially so for a method that ends one’s reproductive capacity. MyDecision/MiDecisión has the potential to address the substantial knowledge gaps about tubal sterilization, including those about its permanence, that prior research has documented.^11,12,13,14^ By providing evidence-based and standardized information directly to patients, this tool has the potential to safeguard against gaps in counseling and against directive counseling toward sterilization. Conversely, there is a robust body of qualitative research highlighting that practitioners do not always honor the choices of people who want to have permanent contraception, often because practitioners do not trust people’s decision-making and worry that they will later regret the procedure.^12,23,24,25,26,27^ Studies have documented that practitioners often dissuade people from sterilization and/or counsel preferentially about long-acting, reversible methods.^26,28^ Patients report that such practices also undermine their autonomy.^12,29^ Overall, having patient-directed tools such as MyDecision/MiDecisión to meet patients’ needs for unbiased and evidence-based information can help ensure informed decision-making and better support people’s reproductive preferences and autonomy. Moreover, the potential of the decision aid to engender higher-quality decisions is particularly relevant following the US Supreme Court decision in *Dobbs v Jackson Women’s Health Organization*, which has led to increased interest in permanent contraception even among younger patients,^30,31,32^ who have been reported to be more likely to experience poststerilization regret.^33,34,35^ The structural shifts in abortion access may precipitate fear-based plans for permanent contraception rather than high-quality decision-making,^36^ amplifying the need for decision support.

Our findings also have important policy implications for the Medicaid regulations governing consent for federally funded sterilization procedures. The current Medicaid regulations were implemented in the 1970s in response to a public outcry about the government’s role in coercive sterilization practices directed at low-income and racial and ethnic minoritized communities.^7^ The regulations require that a standardized Medicaid consent form be signed by the patient at least 30 but no more than 180 days prior to the procedure.^15^ While the intention of the mandatory 30-day waiting period was to ensure voluntary consent, it has inadvertently created a reproductive injustice: patients with Medicaid face numerous barriers to desired sterilization services, with nearly 50% of sterilization requests going unfulfilled.^37^ Thus, numerous advocacy groups and practitioners, including the American Medical Association^38^ and the American College of Obstetricians and Gynecologists,^39,40^ have called for an overhaul of the Medicaid sterilization consent process to better meet the concurrent goals of ensuring informed consent and facilitating equitable access. Specific recommendations include a plain language and simplified consent form^41^ as well as shortening or eliminating the mandatory waiting period.^29,42,43^

Before further consideration is given to eliminating the 30-day waiting period, an evidence-based process for ensuring informed consent is needed as a protective first step given research documenting the shortcomings of the current Medicaid consent process to engender truly informed decision-making.^13,41^ Eliminating the potential for sterilization abuses will require dismantling the complicated and deeply entrenched social and political forces that stratify reproductive value. However, the MyDecision/MiDecisión decision aid offers a scalable safeguard to help ensure that people have complete understanding about the nature of permanent contraception and alternate contraceptive options prior to consenting to the procedure. The current study tested the efficacy of the decision aid in the context of the current Medicaid process, but a future implementation trial could test the decision aid in place of the current Medicaid consent process to offer a more streamlined, evidence-based approach to obtaining consent for permanent contraception procedures.

### Limitations

The current study has a few limitations to consider when interpreting these results. First, this study focused on the efficacy of the decision aid in patients with Medicaid insurance who were contemplating postpartum sterilization—a group that faces relatively greater challenges accessing sterilization information and services. The tool should be tested among other populations, including those who are not currently pregnant and seeking an interval procedure and populations with higher income, to confirm its effectiveness across a broad range of users. Second, the intervention was purposefully delivered earlier in pregnancy at a time when those considering postpartum sterilization are typically deliberating the decision.^17^ However, it is possible that the intervention was delivered prematurely for some participants who were not yet seriously considering permanent contraception and/or had not yet received substantive counseling about postpartum contraceptive options. Our finding that differences in knowledge and decisional conflict between study arms remained statistically significant, although diminished, at the T2 assessment (third trimester), after about 85% of all participants received contraceptive counseling and, on average, 4 months after the intervention was delivered, suggests that the decision aid carried lasting benefits to the time point at which nearly all people have made a sterilization decision.^17^ These findings indicate that when implemented in settings outside of a clinical trial that would enable more flexible access to the tool at the actual time of decision-making, MyDecision/MiDecisión could support high-quality decision-making about sterilization. Finally, our study time frame did not allow us to investigate long-term satisfaction with (or regret about) sterilization decisions. However, decisional conflict has been shown to be strongly correlated with longer-term decisional satisfaction and/or regret.^44,45,46,47^

## Conclusions

This multisite RCT demonstrated the efficacy of MyDecision/MiDecisión to improve sterilization decision-making quality compared with usual care only. This web-based decision aid takes less than 20 minutes to complete and can be widely implemented to supplement practitioner counseling on permanent contraception. Furthermore, if this scalable decision aid is found to be superior to the current Medicaid consent form, it could potentially replace it. Ultimately, once people are safeguarded with an evidence-based process that can truly ensure informed consent, it may be possible to consider potentially shortening or waiving the 30-day waiting period that has restricted access to desired sterilization for many people with low income.

## Notes

Supplement 1.
Trial Protocol

Supplement 2. **eTable 1.** Participant Demographics and Baseline Characteristics **eTable 2.** Evaluation of the Effect of MyDecision/MiDecisión on the Primary Outcomes, by Planned Subgroups

Supplement 3.
Data Sharing Statement
