# Supplementary material for: A Decision Aid to Support Tubal Sterilization Decision-Making Among Pregnant Women: The MyDecision/MiDecisión Randomized Clinical Trial
Source: JAMA Netw Open. 2024 Mar 19;7(3):e242215. doi: 10.1001/jamanetworkopen.2024.2215 (PMC10951734; doi:10.1001/jamanetworkopen.2024.2215)
Supplement: Supplement 1. — Trial Protocol [file jamanetwopen-e242215-s001.pdf]

***MyDecision/MiDecisión*:**

A Decision Support Tool for Women Making Tubal Sterilization Decisions

Study Protocol & Statistical Analysis Plan

Version 3.0, revised January 2024

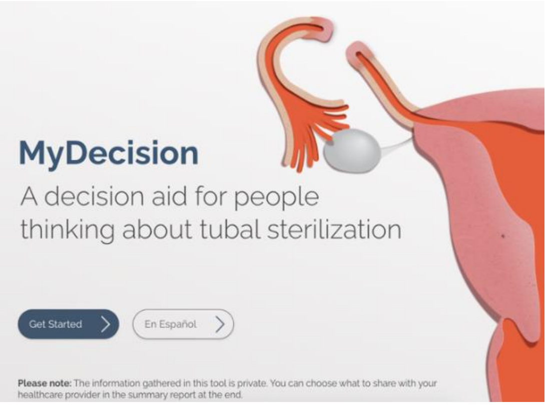

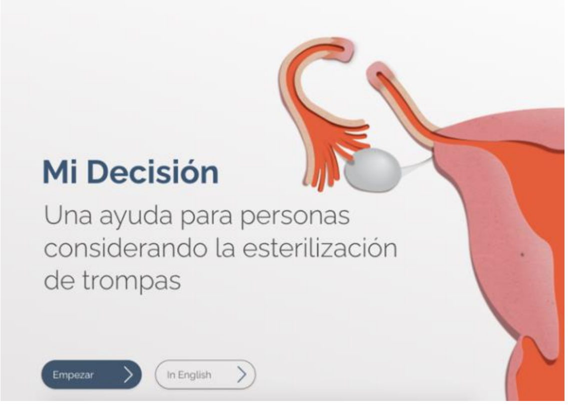

Principal Investigator:
Dr. Sonya Borrero
University of Pittsburgh Department of Medicine

Funded by the National Institute on Minority Health and Health Disparities (NIMHD)
R01MD011678-01

Contents

Study Summary ***3***
Study Objectives ***4***
Hypotheses ***4***
Research Background ***4***
Risk/Benefit Assessment ***4***
Inclusion Criteria ***5***
Recruitment & Screening ***5***
Informed Consent ***7***
Randomization ***7***
Study Arms ***7***
Decision Aid Set-Up & Use ***7***
Intervention and Timepoints ***8***
Withdrawal/Lost to Follow Up ***9***
Participant Reimbursement ***9***
Sample Size Calculation ***9***
Data Collection and Management Responsibilities ***9***
Publication and Data Sharing .... *Error! Bookmark not defined.*
Timeline of Study Activities ***10***
Variables ***12***
Primary Outcome Variables **13**
Secondary Outcome Variables **13**
Exploratory Outcome Measures **14**
Covariates and Baseline Characteristics **18**
Statistical Analyses ***23***
Descriptive and Baseline Statistics **23**
Primary and Secondary Hypotheses **23**
Handling Missing Data **23**
Exploratory Hypotheses **23**
Figure 1. CONSORT Diagram **27**

Study Summary

**Study Title**	"My Decision" Tubal Sterilization Decision Support Tool
**Study Design**	Multi-site randomized clinical trial
**Primary Objective**	To investigate the effect of the *My Decision/Mi Decisión* decision aid on participants' knowledge and decisional conflict regarding tubal sterilization.
**Secondary Objective(s)**	To investigate the effect of the *My Decision/Mi Decisión* decision aid on participants' postpartum contraceptive decision and overall satisfaction with their decision.
**Research Intervention(s)/ Investigational Agent(s)**	*The My Decision/Mi Decisión* decision aid
**Study Population**	Recruitment will occur in Pittsburgh, Pennsylvania; Knoxville, Tennessee; and San Francisco, California. Eligibility criteria includes people who are: 1) <24 weeks pregnant; 2) considering tubal sterilization after delivery; 3) enrolled in or eligible for Medicaid insurance; 4) fluent in English or Spanish; and 5) aged 21-45 years.
**Sites Enrolling Participants**	Magee-Women's Hospital (MWH) in Pittsburgh, Pennsylvania; University of Tennessee Medical Center (UTMC) in Knoxville, TN; Zuckerberg San Francisco General Hospital (ZSFG) in San Francisco, California
**Sample Size**	350 (175 per arm)
**Study Duration for individual participants**	<24 weeks gestation—3 months postpartum (~7-9 months)
**Study Specific Abbreviations/ Definitions**	T1: Time 1 assessment<br>T2: Time 2 assessment<br>T3: Time 3 assessment

Study Objectives

To conduct a **multi-site, randomized controlled trial** (RCT) to test the efficacy of the decision aid plus usual care compared to usual care alone among 350 racially-diverse, low-income women requesting postpartum sterilization with respect to **a)** knowledge, **b)** decisional conflict, **c)** selection of sterilization versus an alternative reversible contraceptive method, and **d)** satisfaction with their decision.

Hypotheses

Primary Hypothesis

Compared to women who receive usual care alone, women randomized to the decision aid arm will have **a)** greater knowledge about sterilization and alternative contraceptive options; **b)** lower decisional conflict.

Secondary Hypotheses

Compared to women who receive usual care alone, women randomized to the decision aid arm will be **a)** less likely to select sterilization and more likely to select an alternative method, and **b)** will be more satisfied with their decision at 3-month postpartum follow-up.

Research Background

Female surgical sterilization is the most commonly used contraceptive method in the US among women 15-49 years, and historically has been disproportionately used by low-income women and women of color.[1] Whether the higher use of sterilization in these populations reflects inappropriate overutilization is unclear. On one hand, low-income and racial minority women frequently misunderstand the permanent nature of sterilization; are often unaware of reversible contraceptive alternatives with similar efficacy, convenience, and safety profiles as sterilization; and commonly experience post-sterilization regret[2–8] – suggesting suboptimal decision making. On the other hand, there is evidence of substantial unmet demand for sterilization among low-income women, due to unique access barriers posed by Medicaid sterilization regulations, putting them at high risk for undesired pregnancy and the adverse health and social consequences associated with unwanted pregnancies and births.[6,9–19]

Medicaid policy currently requires that people requesting a federally-funded sterilization complete a standardized consent form at least 30 days prior to the surgery.[20] This policy was originally instituted in the 1970s to protect vulnerable women from coercive sterilization practices by attempting to ensure informed and voluntary consent.[21,22] However, there is mounting evidence that the consent form is not capable of ensuring patient comprehension, as the reading level is much higher than the average literacy level of the people it intends to serve and misunderstandings about the procedure are common after reviewing the form.[5,23] Furthermore, for many women requesting postpartum sterilization, the mandatory 30-day waiting period and the necessity of successful transfer of the consent form to the delivery suite have created logistical obstacles that preclude them from getting a desired procedure.[6,9–11,13–15] Thus, there is growing consensus that the policy is not only incapable of ensuring informed consent, but also impedes access to desired sterilization for many low income women.[24–32] As the relatively recent alleged coercive sterilizations among female California inmates attest,[33] protection for vulnerable women is still needed. However, given the high rates of unwanted pregnancy among women who request but do not undergo sterilization,[12,34] reducing barriers to desired sterilization is also critical. The lack of a process that meets these two goals – informed consent and timely access – is a critical impediment to progress toward reproductive health equity for low-income women.

Risk/Benefit Assessment

Known Potential Risks

The main potential risk posed by this study is a feeling of discomfort when answering the questions. Due to the nature of our study, contraception, reproductive history and future pregnancy preferences will be discussed. These areas can be sensitive in nature. Our scripts take into consideration these possible discomforts and attempt to mitigate any unintentional harm to participants. Participants will be informed of their right to skip or not answer any questions that cause discomfort.

Another risk of participation is the unlikely breach of information. All of the participant-facing materials will be identified by a unique ID number. All paper study records containing participant identifiers will be stored separately from records identified by the participant's study ID. In addition to the investigators and their research staff, authorized representatives from the University of Pittsburgh Office of Research Protections may review the data solely for the purpose of monitoring

the conduct of this study. De-identified data may also be shared with researchers at the University of Pittsburgh; the University of California, San Francisco; the University of Tennessee, Knoxville; the National Institutes of Health (NIH); and with our decision aid web development team.

The study results will be retained in our research records for at least six years after the study is completed. At that time, the research information will be properly destroyed. If we learn that a participant or someone with them is involved or in serious danger of harm, we will need to inform the appropriate agencies. To help us protect their privacy, we have obtained a Certificate of Confidentiality from the National Institutes of Health. With this certificate, our team cannot be forced to disclose information that may identify a participant, even by court subpoena, in any federal, state, or local civil, criminal, administrative, legislative or other proceedings. The Certificate of Confidentiality will not be used to prevent disclosure to state or local authorities of child abuse, neglect, or harm to self or others.

Known Potential Benefits

There are no known benefits for participants randomized to the control arm of this study. Participants who are randomized to the intervention arm and complete the decision aid may experience benefits including improved knowledge about tubal sterilization, improved knowledge about alternative contraceptive options and improved satisfaction in the contraceptive decision-making processes.

Assessment of Potential Risks and Benefits

This study carries minimal potential risk. Study staff will be provided with scripts that engage the participant in the least discomforting manner and inform them of their right to skip or refuse to answer any question that caused or might cause discomfort. To minimize the potential risk of breach of confidentiality, we will assign an ID number to any identifiable participant data and store these separately from data containing participant identifiers. We conclude that the need for a validated tool that supports informed and value-concordant decision-making regarding tubal sterilization outweighs the overall low risks of participation in our study.

Inclusion Criteria

Women are considered eligible if they are pregnant and <24 weeks gestation; planning to continue their pregnancy; 21-45 years of age; enrolled in or eligible for Medicaid; speak English or, for women recruited in San Francisco, Spanish; and are contemplating sterilization after their delivery. By focusing on women <24 weeks gestation, we hope to capture women who are still deliberating about their post-partum contraception. If the woman is eligible, the Research Assistant reviews the informed consent and receives verbal or written consent from the woman to participate in the study.

Recruitment & Screening

Recruitment

Prior to initiation of study recruitment procedures, healthcare providers at each site will be notified about the study and informed that patients enrolled in the study may be bringing summary sheets generated by the decision aid to clinic visits to facilitate discussions about their postpartum contraceptive decisions or decision-making process. Similar language and procedures will be used to communicate with providers across all sites in advance of the study, including emails and brief presentations during staff meeting times. Of note, the decision aid will not be shown or explained in substantial detail to avoid influencing the standard of care.

Using a combination of in-person and remote methods initiated due to COVID-19), we will **recruit 350 pregnant women** with Medicaid coverage who are contemplating a postpartum sterilization. To **optimize the racial/ethnic and geographic diversity** of our sample, the study will recruit at Magee-Women’s Hospital (MWH) in Pittsburgh, Pennsylvania; University of Tennessee Medical Center (UTMC) in Knoxville, TN; Zuckerberg San Francisco General Hospital (ZSFG) in San Francisco, California. All three sites serve large numbers of low-income women. At these clinical sites, we will employ **recruitment strategies** that we have previously used to successfully recruit pregnant and reproductive-aged women, including posting study flyers throughout each clinic, asking clinical staff to refer potentially eligible women, displaying on message boards and hospital display screens, advertising on Pitt+Me (a University of Pittsburgh research registry), and Craigslist. Pre-COVID, clinic staff were stationed in each of the clinic sites to the clinic waiting rooms to facilitate enrollment during clinical visits.

The COVID-19 pandemic required recruitment redesign. To facilitate remote recruitment in the COVID era, in addition to flyers and message boards, each site will utilize electronic medical records (EMR) to identify potentially eligible persons This process will vary by site based on local IRB and institutional guidelines for research recruitment of patients and are detailed below:

- MWH: Bi-weekly lists of patients enrolled in Medicaid with upcoming 1st or 2nd trimester prenatal visits will be pulled from the EMR and provided to the research team. Each individual on the list will be mailed a letter with study information and instructions to opt-out of being contacted or to contact the team to express interest in participating. After one week, all individuals who did not opt out will be contacted to assess interest and eligibility.
- UTMC: Study staff will review the prenatal clinic schedules each week and filter patients by the study inclusion criteria. Potentially eligible patients may then be approached either in-person during the clinic visit or via telephone to assess interest and eligibility. For in-clinic recruitment, study staff will utilize the healthcare providers to initially approach pre-identified potentially eligible patients to assess interest. If interested and the patient consents, a warm handoff to research staff will occur to assess eligibility.
- ZSFG: study staff will review the prenatal clinic schedules each week and filter patients by the study inclusion criteria. Potentially eligible patients will be called to assess interest and eligibility.

Recruitment Contact Procedures

At Pittsburgh, opt-out letters and a one week waiting period will be required before any recruitment phone calls or emails are attempted. After that, each site will follow the same contact procedures. Contact periods will not exceed 2 weeks since the last successful contact, or in the case of the initial contact, first attempt to contact. Additionally, no more than 6 calls will be attempted during that two-week time frame. For example, if the last contact is a voicemail left by the potential participant expressing interest and a call back, follow up should not exceed 2 weeks from the date of that voicemail. If a potential participant does not show up for a scheduled appointment, follow-up should not exceed 2 weeks from the missed appointment. If the potential participant is reached during initial contact and they request a call back at a later date or time, the two-week time limit and call attempts reset. The preferred timeline for calling follows the rule of 2s: two calls in the morning, two calls in the afternoon, and two calls in the evening or on weekends spread throughout the two-week period to maximize the chance of catching the person at a time when they might be available to answer. No more than 2 emails may be sent and no more than 2 voicemails may be left. Scripts for each interaction and voice messages will be utilized. Texting will only be used for enrolled participants who consent to this method for scheduling future time points or coordinating payment but will not be utilized at the time of recruitment since consent for texting will not have been given at this point.

Screening

For all sites, potential participants that contact the research team or are contacted by the research team either in-person or via phone, mail, email or text message will be given a pre-screening assessment consisting of several questions to ensure they meet basic study eligibility criteria. Written or verbal consent will be obtained to conduct the pre-screening questions. During the pre-screening assessment, interested participants will be given additional study details and an opportunity to ask questions and will also be asked about their access to technology for study participation. If the potential participant is interested in participating in the study, and meets pre-screening eligibility, they will be given the option to participate in the screening, consent, and initial study procedures (randomization, baseline assessment, intervention, and T1 assessment) either in-person or remotely via telephone and/or videoconference.

Due to the evolving nature of the COVID-19 pandemic, in person procedures and safety requirements may vary by site, depending on the current conditions in each region; local restrictions and regulations will be followed. For participants that choose to participate in the study in-person at Pittsburgh and ZSFG, a telephone appointment will be scheduled 24-48 hours before their scheduled in-person healthcare clinic visit. During this telephone appointment, a full study screening and COVID-19 screening will be conducted. If the potential participant meets full study eligibility criteria, and verbally screens negative for COVID-19 symptoms and risk factors, research staff will meet the participant before or after the participant’s in-person healthcare appointment at their local study site (i.e., Magee Women's Hospital or McKee Place for Pittsburgh participants, and Zuckerberg San Francisco General Hospital for San Francisco participants) to complete the written informed consent for enrollment and the Baseline and T1 interviews.

Since study staff at UTMC are still recruiting in-person (unlike MWH and ZSFG which are using entirely remote recruitment procedures), patients at UTMC will have completed COVID-19 screenings during their regularly scheduled visit and will not have to complete a separate COVID screening by study staff. If a potential participant is recruited in-person but does not have time to complete all of the screening, consent, and initial study activities (randomization, baseline assessment, intervention, and T1 assessment) at that time, research staff should conduct the pre-screening assessment and verbal consent to collect contact information and set up another appointment. If a potential participant is recruited in-person, and they express interest in the study but do not have time for any assessment including pre-screen questions, study staff may ask for verbal consent to collect contact information. During the follow-up call, study staff should complete the pre-screen assessment and, if preliminarily eligible, schedule an appointment for the rest of the initial study activities.

For participants that choose to participate remotely, a telephone and/or videoconference appointment will be scheduled and conducted at the participant's convenience. During this appointment, a full screening will be conducted to ensure the participant is still eligible. Verbal consent will be obtained to conduct the full screening questions over the phone. Informed consent, baseline assessment, the intervention, and Time 1 assessment will then be conducted remotely over the phone or videoconference.

Informed Consent

The informed consent form is written in accordance with each study site's Institutional Review Board guidelines. Participants will be given an overview of the study, as well as details about time commitments, risks and benefits, confidentiality, and compensation. A copy of the written consents will be given at the time of signing and a copy of verbal consent scripts will be sent to the participant's email at the conclusion of their remote visit.

Randomization

Randomization is stratified by site and occurs at the patient level to allocate participants to each of the 2 study arms in a 1:1 ratio, using permutated block randomization that is integrated into the web-based data collection system.

Study Arms

Usual care arm

Our control condition is usual care. Usual care sterilization counseling is likely quite variable, but one consistency is that for women requesting a Medicaid-funded postpartum sterilization, providers must ensure that the standardized Medicaid consent form is signed at least 30 days but no more than 180 days prior to delivery. Consequently, most providers complete the Medicaid sterilization consent form with their patients between 24-32 weeks gestation. This process typically includes counseling on all forms of contraception.

Intervention arm

Participants randomized to the intervention arm use our novel web-based tool prior to 24 weeks gestation. As all women requesting a Medicaid-funded procedure must adhere to the federal consent process, women in the intervention arm who remain interested in undergoing sterilization as they proceed through pregnancy must complete the consent process as per usual care. Thus, our intervention arm is decision aid plus usual care.

Decision Aid Set-Up & Use

Remote Participation

- The tool can be accessed on a smartphone, tablet, or computer. Research assistants will instruct the participant to go to the provided URL (sent via email or text) and enter their participant ID and access code to enter the tool. Study staff will provide them with the link, participant ID, and access code. Participant IDs for the tool will be identical to their REDCap IDs for ease of connecting the data during analysis.
- Users are expected to complete the tool in a single session.
- Users will have the option to quit the tool and delete their cached data at any time. This should be communicated to participants, so that they know they have this option if using a shared device. They can use this feature by clicking the "End Session" button that they can access from a dropdown carrot at the top right of the display.
- Participants in the trial will only be able to access and complete the tool once. Once completed, the login information provided will no longer enable them to login. This ensures the data is protected from accidental changes or re-visiting the information at a later time.

Study-Owned iPad for In-Person Participation

- Logging a participant into the tool is exactly the same procedure as described above for remote participation, but the study staff can complete the login or can instruct the participant on how to access it while in the room together.
- After completion of the tool, study staff can print the summary report and provide it to the participant. Study staff then should use the "End Session" button to clear the cache to ensure privacy of data collected. Selecting this will erase the data on the local device, clearing the log in and the participant's responses from the browser's cache.

Intervention and Timepoints

Baseline, Intervention, and Time 1 Assessment

Immediately (when possible) after consent, study staff will conduct baseline interviews to assess patient demographic characteristics as well as medical and reproductive history. All assessments will be administered as interviews by study staff who enter the data into REDCap. Women will then be randomized to one of the 2 study arms using computer-generated allocation that is incorporated into the study database. Those randomized to the control (usual care) condition will immediately complete their Time 1 assessment (in person or via phone) to assess knowledge, contraceptive method selected, and decisional conflict. After the baseline survey, those randomized to the intervention arm immediately will use the decision aid on a phone, tablet, or computer. Participants randomized to the decision aid will receive a summary sheet (via email or printout) of their responses, which they are encouraged to share with their healthcare provider. Study staff members will be available throughout to assist participants as needed to answer questions. Following completion of the intervention, staff will then conduct the T1 assessment with the participants. Study procedures at this initial time point will take a total of 60-90 minutes for women in the intervention group and 30-45 minutes for those in the usual care arm.

Time 2 Assessment

Study staff members will conduct Time 2 (T2) assessments by telephone between 32-36 weeks gestation to re-assess knowledge, contraceptive method selected, and decisional conflict closer to delivery and after the Medicaid sterilization consent form is typically signed. T2 follow-up contact will not exceed 4 weeks from the date of the first contact attempt. A maximum of 5 messages will be left on voicemail (2 during the first week of follow-up and one per week for remaining weeks), unless contact is established with the participant and call backs are requested. The same procedure should be followed for email. The research team will attempt to reach participant for T3 even if they did not complete T2, as long as they did not explicitly withdraw from the study.

Time 3 Assessment

The final Time 3 (T3) assessment will also be conducted via telephone 3 months postpartum to determine which method, if any, women are actually using and satisfaction with their decision. T3 follow-up contact will not exceed 4 weeks from the date of the first contact attempt and follows the same standards for maximum attempts of contact as T2. As the 3-month follow-up is likely too proximal to experience regret for sterilization decisions, we will focus on other widely-used constructs (decisional conflict and satisfaction with decision) that are strongly correlated with decisional regret.[35,36]

For those participants whose pregnancy ends before T2 due to miscarriage, termination, or stillbirth, the research team will only proceed with T3 if the participant wishes to continue with their participation. If the participant agrees to be contacted for a T3 interview, T3 follow-up contact will begin at least 3 months after the date the pregnancy ended.

Figure: Schematic representation of study

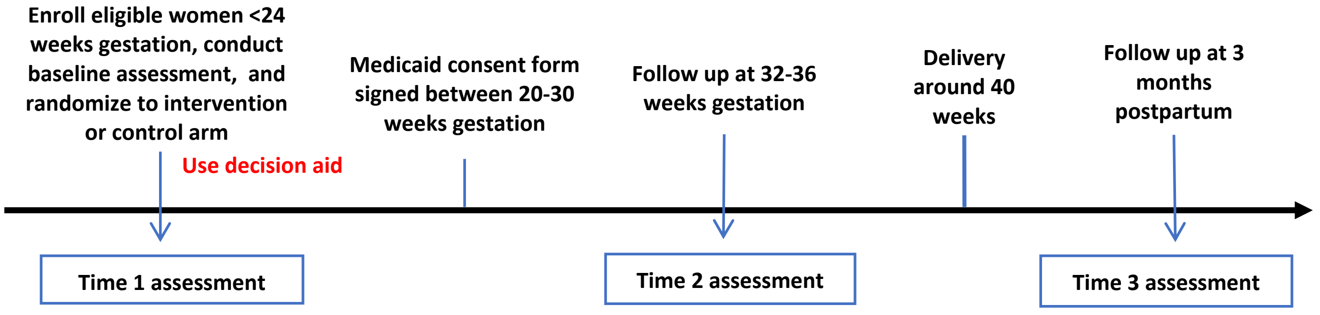

Withdrawal/Lost to Follow Up

Withdrawal

A participant may withdraw from the study at any time and for any reason. They simply have to communicate to the research staff that they no longer wish to participate in the study.

Lost to Follow Up

Participants can be deemed lost to follow-up in two scenarios:

1. Once a participant has signed the consent form during their T1 appointment, they are considered “Enrolled” in this study. If a participant has been enrolled but does not complete their T1 appointment during a single session, we refer to these cases as “partial T1s.” If a partial T1 is unable to be rescheduled to complete their interview and if they do not respond to contact attempts made by research staff, they may be classified as Lost to Follow Up. [Contact attempts should be conducted according to outlined follow-up procedures above. Contact attempts may cease after 4 weeks have elapsed from missed appointment with no response from participant OR if increased gestational age would place participant outside of eligibility window for T1.]
2. If the research team is unable to reach a participant at **Time 3** after follow up protocols are followed as specified, participant may be deemed Lost to Follow Up.

NOTE: If a participant is unable to be reached at Time 2, they are not considered lost to follow-up and the research team should attempt to reach them again at T3. For these cases, Research staff should have this documented in their records, and then follow up procedures for T3 should be followed as specified above.

Participant Reimbursement

Participants will be reimbursed up to $90 for time dedicated to participating in the study. The breakdown for payments is $40 for T1, $20 for T2, and $30 for T3. We have considered both the time it takes to complete each study visit as well as appropriately incentivizing longevity for retention purposes. Pittsburgh and Tennessee will utilize a payment system that provides reloadable debit/Mastercards for participants. These cards will be provided either in-person or mailed once T1 is completed. Once participants have possession of the card, all payments for study assessments can be made remotely. Sites in San Francisco will utilize gift cards that can be mailed, emailed, or sent via text to participants at the completion of each study time point.

Sample Size Calculation

We based our sample size on the standardized mean differences (Cohen’s d) for the co-primary outcomes of knowledge (0.46) and decisional conflict (0.36) reported in the 2014 Cochrane review of decision aid RCTs. Assuming two-sided alpha levels of 0.025 (for each co-primary outcome) and 15% attrition, 175 women per arm afforded us 80% power to detect these differences.[37]

Data Collection and Management Responsibilities

Data Management

Research staff are responsible for maintaining the confidentiality of study participants. All of the participant-facing materials will be identified by a unique ID number. All paper study records containing participant identifiers will be stored separately from records identified by the participant’s study ID. Any files linking a participant’s ID number with their name will be stored on each site’s hard drive, which is only accessible through two-factor authentication. All other study data will be collected and managed using REDCap electronic data capture tools hosted by the University of Pittsburgh. REDCap (Research Electronic Data Capture) is a secure, web-based software platform designed to support data capture for research studies, providing 1) an intuitive interface for validated data capture; 2) audit trails for tracking data manipulation and export procedures; 3) automated export procedures for seamless data downloads to common statistical packages; and 4) procedures for data integration and interoperability with external sources.

In addition to the investigators and their research staff, authorized representatives from the University of Pittsburgh Office of Research Protections may review the data solely for the purpose of monitoring the conduct of this study. De-identified data may also be shared with researchers at the University of Pittsburgh; the University of California, San Francisco ; the

University of Tennessee, Knoxville, the National Institutes of Health (NIH); and with our decision aid web development team.

Table: Maintenance and location of data sources

Data:	Data Location:	Task:	When to Perform Task:	Notes:
Clinic patient schedules	EMR database	Review clinic schedules to screen for potentially eligible patients	Weekly or biweekly	
Patient contact information	EMR database	After applying eligibility criteria, store on REDCap & site hard drive	Each contact with patient	
Consent forms	Verbal Consents are stored in research assistant's laptop. Written Consents are stored in a study binder behind two locks accessible to only study staff	Save or scan and upload to REDCap & site-specific hard drive	During study enrollment	Verbal Consents: Provide participant with blank copy (if possible) Written Consents: Provide copy of signed consent to participant
Decision aid user data	WordPress & Google Analytics			Only unique user IDs will be stored
Other data (screening, post-intervention, T2 and T3 surveys)	RED Cap	Log enrollment status, T1-T3 completion dates in site hard drive	When new data is available	No hardcopies will be produced

Quality Control Measures

Research assistants at all sites will be responsible for the following QC measures:

- Contacting all participants who have missed their study appointment.
- Reminding participants of their study appointments, regardless of mode of interview (remote or in-person).
- Ensuring that each data field is filled out appropriately. If a participant does not wish to disclose something, then the research assistant should document this.

;

This study will be conducted in accordance with the following publication and data sharing policies and regulations:

National Institutes of Health (NIH) Public Access Policy, which ensures that the public has access to the published results of NIH funded research. It requires scientists to submit final peer-reviewed journal manuscripts that arise from NIH funds to the digital archive PubMed Central upon acceptance for publication.

This study will comply with the NIH Data Sharing Policy and Policy on the Dissemination of NIH-Funded Clinical Trial Information and the Clinical Trials Registration and Results Information Submission rule. As such, this trial will be registered at ClinicalTrials.gov, and results information from this trial will be submitted to ClinicalTrials.gov. In addition, every attempt will be made to publish results in peer-reviewed journals. Data from this study may be requested from other researchers after the completion of the primary endpoint by emailing mydecisionstudy@pitt.edu.

Timeline of Study Activities

Aim 3: RCT to Test Decision Aid Efficacy

	Year 1: 5/1/19-4/30/20				Year 2: 5/1/20-4/30/21				Year 3: 5/1/21-4/30/22				Year 4: 5/1/22-4/30/23				Year 5: 5/1/23-4/30/24			
Start-up (finalize materials, IRB approval at all sites)																				
Participant recruitment				COVID DELAYS																
3-month post-partum follow-up																				
Data analysis and Dissemination																				

Variables

Variable	BL	T1	T2	T3
**Primary Outcomes**				
Participant knowledge about tubal sterilization		X	X	
Participant decisional conflict in postpartum contraceptive choice		X	X	
**Secondary Outcomes**				
Contraceptive method selected		X	X	
Participant satisfaction with contraceptive decision making				X
**Exploratory Outcomes**				
Desire for sterilization*		X		
Contraceptive method used				X
Ease in contraceptive decision-making		X	X	
Certainty about sterilization decision		X	X	
Participant satisfaction with current contraceptive method				X
Participant confidence with contraceptive method(s)				X
Subsequent pregnancy				X
Participant satisfaction with sterilization counseling				X
Satisfaction with, Recommendations for, and Learning from the Decision Aid**		X		
Proportion of intervention group participants who shared the summary sheet with their provider			X	
Proportion of those who signed Medicaid waiver but did not receive sterilization (calculated)				X
Reason for signing consent form but not having sterilization				X
Reason for not having sterilization (mismatch T2-T3)				X
Reason for not having other contraceptive method (mismatch T2-T3)				X
Shared summary sheet with provider**			X	X
**Covariates and Baseline Characteristics**				
Future Pregnancy Desires: Do you want to get pregnant in the future?*		X		
Intendedness of Current Pregnancy				
Right before you got pregnant with this pregnancy, did you want to have a baby at any time in the future?	X			
Would you say you got pregnant sooner, about the right time, or later than you wanted?	X			
On a scale of 0-10, where 0 means you were very unhappy to be pregnant and 10 means that you were very happy to be pregnant, tell me which number best describes how you felt when you found out you were pregnant?	X			
On a scale of 0-10, where 0 means trying hard not to get pregnant, and 10 means trying hard to get pregnant, if you had to rate how much you were trying to get pregnant or avoid pregnancy before you got pregnant, how would you rate yourself?	X			
Signed Medicaid consent	X		X	X
Desire for sterilization*		X		
Thoughts of sterilization before (and reasons for not)	X			
Received any provider contraceptive counseling	X		X	
Received any provider sterilization counseling during current pregnancy	X		X	X
Received any provider sterilization counseling during a prior pregnancy	X			
Age	X			
Race/ethnicity	X			
Relationship status	X			
Gravida	X			
Parity	X			
History of abortion	X			
Medical and mental health history	X			
BMI	X			
Gestational age (less than 24 weeks or not)	X			
Medicaid coverage of the pregnancy	X			
Religion	X			
Highest education	X			
Health literacy	X			
Income/# people in household/federal poverty level	X			
Medicaid status	X		X	X
Contaceptive method history	X			
In-person or virtual delivery of the Decision Aid				
Language of the decision aid	X			

*Notes: *this was measured before and after the decision aid for intervention group at at the very beginning of T1 assesssment for control group; **this was only measured for the intervention group*

Primary Outcome Variables

Outcome	Description of Data Collected	Time Point	Variable Structure	Format for Data Analysis/ Transformations
Participant Knowledge about Tubal Sterilization	Participant responses to 10 True/False items adapted from previously published studies of tubal sterilization knowledge and informed by in-depth interviews with women about sterilization decision making.[*] Items assess knowledge about tubal sterilization and alternative contraception options and will be coded as correct vs. incorrect, with "Don't Know" being coded as an incorrect response. The percentage of correct responses across the 10 questions will be calculated for each participant. The score range is 0 to 100%.	T1: less than 24 weeks gest.	Continuous (0-100%)	Continuous (0-100%)
Participant Decisional Conflict in Postpartum Contraceptive Choice	Assessed by participant responses to the low literacy version of the Decisional Conflict Scale (DCS). The DCS is a validated measure to assess participants' decisional conflict in medical decision-making. The DCS low literacy version includes 10 items about experience of conflict, with "Yes" = 0, "unsure" = 2 and "No" = 4. All 10 items are a) summed; b) divided by 10; and c) multiplied by 25. Scores range from 0 (no decisional conflict) to 100 (extremely high decisional conflict). †	T1: less than 24 weeks gest.	Continuous (0-100)	Continuous (0-100)

*Borrero S, Abebe K, Dehlendorf C, et al. Racial variation in tubal sterilization rates: role of patient-level factors. *Fertil Steril*. 2011;95(1):17-22. doi:10.1016/j.fertnstert.2010.05.031
†User manual - decisional conflict scale. https://decisionaid.ohri.ca/docs/develop/User_Manuals/UM_Decisional_Conflict.pdf. Published 1993. Accessed August 8, 2022.

Secondary Outcome Variables

Outcome	Description	Time Point	Variable Structure	Structure for Analysis
Contraceptive Method Selected	Will determine the proportion of women who select sterilization versus another method of contraception at Time 1. Assessed by participant responses to an item asking which contraceptive method they are planning to use after delivery.<br><br>Note: for participants who give multiple methods as their response, they are asked: "which method are you most leaning towards using after the birth of your baby?"	T1: less than 24 weeks gest.	Categorical (proportions by category)	Dichotomous (proportion sterilization vs. all other categories)<br><br>*Use the primary method identified if participant selects multiple methods
Participant Satisfaction with Decision Making	Assessed by participant responses to the Satisfaction with Decision Scale (SWD), a validated 6-item scale measuring participants' satisfaction with healthcare decision making. * The SWD uses a 5-point Likert scale where 1 is "Strongly Disagree" and 5 is "Strongly Agree." A summary score is calculated as an average of all items with a range of 1-5, with a higher score reflecting a higher level of satisfaction with the decision.	T3: 3 months postpartum	Continuous (1-5)	Continuous (1-5)

*Holmes-Rovner M, Kroll J, Schmitt N, et al. Patient satisfaction with health care decisions. *Medical Decision Making*. 1996;16(1):58-64. doi:10.1177/0272989x9601600114

Exploratory Outcome Measures

Outcome	Description	Time Point	Variable Structure	Structure for Analysis
Desire for sterilization	At this point, how do you feel about sterilization? 5-point Likert (I definitely don’t want sterilization, I probably don’t want sterilization, neutral/not sure, I probably do want sterilization, I definitely do want sterilization*	T1 for controls + before and after Decision Aid for intervention group	Ordinal (5-point Likert)	This can be measured two ways:<br><br>1) Continuous (1-5)<br>2) Ordinal:<br>Definitely don’t (1)à5<br>Probably don’t (2) à 4<br>Not sure (3) = 3<br>Probably do (4)<br>Definitely do (5)<br>*Where 3= unsure, 4=probably, 5=definitely*<br><br>Pre-post changes for intervention group<br><br>Post-DA for intervention group vs T1 for controls
Participant Knowledge about Tubal Sterilization	Participant responses to 10 True/False items adapted from previously published studies of tubal sterilization knowledge and informed by in-depth interviews with women about sterilization decision making. Items assess knowledge about tubal sterilization and alternative contraception options and will be coded as correct vs. incorrect, with "Don't Know" being coded as an incorrect response. The percentage of correct responses across the 10 questions will be calculated for each participant. The score range is 0 to 100%. Participant knowledge will be measured at Time 2 to assess the sustained impact of the decision aid on knowledge.	T2: 32-36 weeks gestation	Continuous (0-100%)	Continuous (0-100%)
Participant Decisional Conflict in Postpartum Contraceptive Choice	Assessed by participant responses to the low literacy version of the Decisional Conflict Scale (DCS). The DCS is a validated measure to assess participants' decisional conflict in medical decision-making. The DCS low literacy version includes 10 items about experience of conflict, with "Yes" = 0, "unsure" = 2 and "No" = 4. All 10 items are a) summed; b) divided by 10; and c) multiplied by 25. Scores range from 0 (no decisional conflict) to 100 (extremely high decisional conflict). Decisional conflict will be measured at Time 2 to assess the sustained impact of the decision aid on decisional conflict.	T2: 32-36 weeks gestation	Continuous (0-100)	Continuous (0-100)
Contraceptive Method Selected	Will determine the proportion of women who select sterilization versus another method of contraception at Time 2. Assessed by participant responses to an item asking which contraceptive method they are planning to use after delivery.	T2: 32-36 weeks gestation	Categorical (proportions by category)	Dichotomous<br>(proportion sterilization vs. all other categories)
Contraceptive Method(s) Used	Assessed by participant responses to a multiple choice item asking which methods of contraception they have used or received since their pregnancy ended.<br><br>¡ Birth Control Pills<br>¡ Male Condom<br>¡ Partner’s Vasectomy<br>¡ Tubal sterilization<br>¡ Withdrawal or pulling out<br>¡ Contraceptive injection (Depo-Provera)<br>¡ Hormonal Implant (Nexplanon)<br>¡ Natural family planning/rhythm method	T3: 3 months postpartum	Categorical (proportions by category)	All participant: Dichotomous (proportion sterilization vs. all other categories)<br><br>Among women who wanted sterilization: Dichotomous (proportion sterilization vs. all other categories)

	¡ Female condom<br>¡ Sponge,diaphragm, or cervical cap<br>¡ Emergency contraception<br>¡ Spermicides<br>¡ Intrauterine device (IUD)<br>¡ Contraceptive patch<br>¡ Vaginal contraceptive ring (Nuva Ring)<br>¡ Other (*Specify*): _________________<br>¡ None<br>¡ Don’t know or unsure			
Ease in Contraceptive Decision Making	Assessed by participant responses to a 10 point scale about ease of decision making with regards to contraceptive method selection. Response options range from 0 ("Strongly Disagree" that this is an easy decision to make) to 10 ("Strongly Agree" that this an easy decision to make").*	T1: less than 24 weeks gest.<br><br>T2: 32-36 weeks gestation	Ordinal (1-10 Likert)	Continuous (1-10)
Certainty about Tubal Sterilization Decision	Assessed by participant responses to a 10 point scale indicating certainty in desire to obtain or not obtain a tubal sterilization. Response options range from 0 ("Very Certain that I Do Not Want") to 10 ("Very Certain that I Do Want").*	T1: less than 24 weeks gest.<br><br>T2: 32-36 weeks gestation	Ordinal (1-10 Likert)	We can analyze this 3 ways:<br><br>1) Nominal: 1-3 score=1 (certain about not getting it), 4-7 score=2 (uncertain), 8-10 score=3 (certain about getting it)<br><br>2)Continuous (5-10) with recoding to measure only certainty and not whether it’s a certain yes or certain no:<br>0-->10<br>1--> 9<br>2-->8<br>3-->7<br>4-->6<br>5=5<br><br>3) Dichotomous: 1-3 and 8-10= 1 (certain) and 4-7=0 (uncertain)
Participant Satisfaction with Current Contraceptive Method(s)†	Assessed by participant responses to a 5-point Likert scale regarding satisfaction with current contraceptive method(s). Response options range from 1 ("Very Unsatisfied") to 5 ("Very Satisfied").*	T3: 3 months postpartum	Ordinal (1-5 Likert)	Continuous (1-5)
Participant Confidence in Contraceptive Method Selection	Assessed by participant responses to a 5-point Likert scale regarding participant confidence that the method(s) is/are "right for me". Response options range from 1 ("Not at All Confident") to 5 ("Completely Confident").	T3: 3 months postpartum	Ordinal (1-5 Likert)	Continuous (1-5)
Subsequent Pregnancy Since Delivery	Assessed by participant-reported survey responses to a Yes/No item about whether a pregnancy has occurred since delivery.	T3: 3 months postpartum	Dichotomous (yes/no)	Dichotomous (yes/no)
Participant Satisfaction with Sterilization Counseling	Participant responses to an 8-item patient-provider communication scale created by the PI to assess participant satisfaction with the sterilization counseling experience. Response options range from 1 ("Strongly Disagree") to 5 ("Strongly Agree"). Items will be examined individually. In addition, a summary score will be created with items 2,3,6 reverse coded such that the range will be 8-40 with a higher score reflecting greater satisfaction with sterilization counseling	T3: 3 months postpartum	Continuous (8-40)	Continuous (8-40)

Overall satisfaction with decision tool	Overall I like the "My Decision" tool	T1 for intervention group only	Ordinal (5-point Likert)	Continuous (1-5)<br><br>Also: dichotomous (Agree/Strongly Agree vs. all other categories)
Recommend tool	"I would recommend the tool to other women"	T1 for intervention group only	Ordinal (5-point Likert)	Continuous (1-5)<br><br>Also: dichotomous (Agree/Strongly Agree vs. all other categories)
Learned from tool	I learned new information from the tool	T1 for intervention group only	Ordinal (5-point Likert)	Continuous (1-5)<br><br>Also: dichotomous (Agree/Strongly Agree vs. all other categories)
Information amount in tool	The amount of information in the tool was...	T1 for intervention group only	Nominal (too much, too little, just right)	Nominal (1=too much 2= too little, 3= just right)
Device used to complete the decision aid	What device did you use to complete the decision aid?	T1 for intervention group only	Nominal (smartphone, ipad/tablet, desktop/laptop computer)	Nominal (1=smartphone, 2=ipad/table, 3= desktop/laptop computer)
Comments about tool experience	Do you have any comments about your experience using the tool?	T1 for intervention group only	Dichotomous (Yes-please specify, No)<br><br>Open-ended "please specify" responses	Limit this analysis to only those who answer Yes<br><br>Content analysis and categorization of responses
Shared summary sheet with their provider by T2/T3	After you completed the "My Decision" decision aid, you received a summary sheet. Did you ever share the summary sheet with your healthcare provider?	T2<br><br>Again asked at T3 if participant said "No" at T2	Nominal: Yes<br>No<br>Don't Know	Dichotomous (yes/no) [exclude Don't Know]
CALCULATED: Proportion of those who signed Medicaid waiver but DID NOT receive sterilization by T3‡	Limit to the subgroup who signed the Medicaid sterilization waiver and consent:<br>"Medicaid requires that women who desire a tubal sterilization sign a consent form at least 30 days before undergoing a tubal sterilization procedure. Have you signed the Medicaid tubal sterilization consent form during this pregnancy?"<br><br>Of those who signed the consent, what proportion did NOT receive sterilization services as determined by participant responses to a multiple choice item asking which methods of contraception they have used or received since their pregnancy ended.<br><br>Denominator Description:	Medicaid sterilization consent signed: Asked at Baseline, T2, T3<br><br>Sterilization services NOT received: T3	Medicaid sterilization consent signed:<br>¡ Yes<br>¡ No<br>¡ Don't Know<br><br>Sterilization services NOT received: multiple variables, each dichotomous	CALCULATED AS:<br>-among those who signed the Medicaid waiver (YES at BL, T2, or T3), what proportion DID NOT receive sterilization (operationalized as "No" on the T3 contraceptive method used question answer choice "tubal sterilization"
Reasons why sterilization consent signed but no sterilization procedure completed‡	Can you tell me a little bit about why you signed the Medicaid consent form, but did not go through with the tubal sterilization procedure?	T3	Open-ended "please specify" responses	Content analysis and categorization of responses using proportions
Reason for mismatch between non-tubal sterilization contraceptive method	Can you tell me a little bit about why you are using a different method of	T3	Open-ended	Content analysis and categorization of responses

preferred at T2 and used at T3‡	contraception now? OR Can you tell me a little bit about why you are not using birth control? (Probe as necessary: Why did you change your mind, did you face any barriers?)			
Medical concerns	For people who reported at T2 wanting a method *other than sterilization* but their current contraception doesn't match	T3	Dichotomous (yes/no)	Dichotomous (yes/no)
Financial concerns	For people who reported at T2 wanting a method *other than sterilization* but their current contraception doesn't match	T3	Dichotomous (yes/no)	Dichotomous (yes/no)
Method was unavailable/too difficult to obtain	For people who reported at T2 wanting a method *other than sterilization* but their current contraception doesn't match	T3	Dichotomous (yes/no)	Dichotomous (yes/no)
You were concerned about the side effects	For people who reported at T2 wanting a method *other than sterilization* but their current contraception doesn't match	T3	Dichotomous (yes/no)	Dichotomous (yes/no)
Method was too difficult to use	For people who reported at T2 wanting a method *other than sterilization* but their current contraception doesn't match	T3	Dichotomous (yes/no)	Dichotomous (yes/no)
You were concerned about its effectiveness/it would not prevent pregnancy	For people who reported at T2 wanting a method *other than sterilization* but their current contraception doesn't match	T3	Dichotomous (yes/no)	Dichotomous (yes/no)
Your partner did not like this method	For people who reported at T2 wanting a method *other than sterilization* but their current contraception doesn't match	T3	Dichotomous (yes/no)	Dichotomous (yes/no)
This method did not prevent STIs	For people who reported at T2 wanting a method *other than sterilization* but their current contraception doesn't match	T3	Dichotomous (yes/no)	Dichotomous (yes/no)
Something else	For people who reported at T2 wanting a method *other than sterilization* but their current contraception doesn't match	T3	Dichotomous (yes/no)	Dichotomous (yes/no)
Reason for mismatch between preferring sterilization at T2 and not having used tubal sterilization at T3‡	Can you tell me a little bit about why you are using a different method of contraception now? OR Can you tell me a little bit about why you are not using birth control? (Probe as necessary: Why did you change your mind, did you face any barriers?)	T3	Open-ended	Content analysis and categorization of responses
Medical concerns	For people who reported at T2 *wanting sterilization* as their method but did not undergo sterilization	T3	Dichotomous (yes/no)	Dichotomous (yes/no)
Financial concerns	For people who reported at T2 *wanting sterilization* as their method but did not undergo sterilization	T3	Dichotomous (yes/no)	Dichotomous (yes/no)
The consent form was not completed on time or in its entirety	For people who reported at T2 *wanting sterilization* as their method but did not undergo sterilization	T3	Dichotomous (yes/no)	Dichotomous (yes/no)
The operating room was not available	For people who reported at T2 *wanting sterilization* as their method but did not undergo sterilization	T3	Dichotomous (yes/no)	Dichotomous (yes/no)
The wait between your delivery and getting the procedure was too long	For people who reported at T2 *wanting sterilization* as their method but did not undergo sterilization	T3	Dichotomous (yes/no)	Dichotomous (yes/no)
You decided you wanted a reversible method of contraception	For people who reported at T2 *wanting sterilization* as their method but did not	T3	Dichotomous (yes/no)	Dichotomous (yes/no)

	undergo sterilization			
You were worried about getting anesthesia	For people who reported at T2 *wanting sterilization* as their method but did not undergo sterilization	T3	Dichotomous (yes/no)	Dichotomous (yes/no)
You were concerned about the recovery process	For people who reported at T2 *wanting sterilization* as their method but did not undergo sterilization	T3	Dichotomous (yes/no)	Dichotomous (yes/no)
Other reason	For people who reported at T2 *wanting sterilization* as their method but did not undergo sterilization	T3	Open-ended	Content analysis and categorization of responses

*Hamed Taherdoost. What Is the Best Response Scale for Survey and Questionnaire Design; Review of Different Lengths of Rating Scale / Attitude Scale / Likert Scale. *International Journal of Academic Research in Management (IJARM)*, Helvetic Editions, 2019, 8. ffhal-02557308f

†Committee opinion no. 587. *ACOG Committee Opinion*. 2014;123(2):389-393. doi:10.1097/01.aog.0000443279.14017.12Hahn TA, McKenzie F, Hoffman SM, Daggy J, Tucker Edmonds B. A prospective study on the effects of Medicaid regulation and other barriers to obtaining postpartum sterilization. *Journal of Midwifery & Women's Health*. 2018;64(2):186-193. doi:10.1111/jmwh.12909

‡ Hahn TA, McKenzie F, Hoffman SM, Daggy J, Tucker Edmonds B. A prospective study on the effects of Medicaid regulation and other barriers to obtaining postpartum sterilization. *Journal of Midwifery & Women's Health*. 2018;64(2):186-193. doi:10.1111/jmwh.12909

Covariates and Baseline Characteristics

Covariate	Description	Time Point	Variable Structure	Structure for Analysis
Future pregnancy desires	Do you want to get pregnant in the future?"<br><br>Note: this was measured in the decision aid for intervention group and at the very beginning of T1 assessment for control group	BL	Ordinal (1-5)<br><br>Definitely no (1)<br>Probably no (2)<br>Not Sure/Fine Either Way (3)<br>Probably Yes (4)<br>Definitely Yes (5)	Ordinal (1-5)<br><br>Definitely no (1)<br>Probably no (2)<br>Not Sure/Fine Either Way (3)<br>Probably Yes (4)<br>Definitely Yes (5)
Intendedness of Current Pregnancy*	Unintended= Unwanted (did not want baby then or in future) or got pregnant sooner than wanted	BL	See below	See below
	Unwanted pregnancy= Right before you got pregnant with this pregnancy, did you want to have a baby at any time in the future?	BL	Dichotomous (yes/no)	Dichotomous (yes/no)
	Mistimed= Would you say you got pregnant sooner, about the right time, or later than you wanted?<br><br>-only asked of participants who said "Yes" to "did you want to have a baby at any time in the future?"	BL	Nominal:<br>¡ Sooner<br>¡ Right time<br>¡ Later<br>¡ Didn't care/ Don't know	Dichotomous "Too Soon"<br><br>1=sooner, 0= right time, 0= later, 0= didn't care/don't know
	On a scale of 0-10, where 0 means you were very unhappy to be pregnant and 10 means that you were very happy to be pregnant, tell me which number best describes how you felt when you found out you were pregnant?	BL	Continuous (1-10 Likert)	Continuous (1-10 Likert)
	On a scale of 0-10, where 0 means trying hard not to get pregnant, and 10 means trying hard to get pregnant, if you had to rate how much you were trying to get pregnant or avoid pregnancy before you got pregnant, how would you rate yourself?	BL	Continuous (1-10 Likert)	Continuous (1-10 Likert)
Signed Medicaid Consent†	Medicaid requires that women who desire a tubal sterilization sign a consent form at least 30 days before undergoing a tubal sterilization procedure. Have you signed the Medicaid tubal sterilization consent form during this pregnancy?	Baseline, T2. T3	¡ Yes<br>¡ No<br>¡ Don't Know	Categorical: 0=no, 1= yes, 2= don't know

Desire for sterilization	At this point, how do you feel about sterilization? 5-point Likert (I definitely don't want sterilization, I probably don't want sterilization, neutral/not sure, I probably do want sterilization, I definitely do want sterilization	T1 for controls + before Decision Aid for intervention group	Ordinal (5-point Likert)	This can be measured two ways:<br><br>1) Continuous (1-5)<br>2) Ordinal:<br>Definitely don't (1)<br>Probably don't (2)<br>Not sure (3) = 3<br>Probably do (4)<br>Definitely do (5)<br><br>Group differences at baseline (pre-DA for intervention group, T1 for control)
Thoughts of sterilization and reasons not used	Have you ever thought about having a tubal sterilization before, but then did not get one?<br><br>If yes, why didn't you get a tubal sterilization?	Baseline	Dichotomous (yes/no)<br><br><br>Open-ended	Dichotomous (yes/no)<br><br><br>Content analysis and categorization of responses
Provider contraceptive counseling:	Have you received any counseling from a healthcare professional about contraception or your birth control options after your delivery?	BL, T2	Dichotomous (yes/no)<br><br>Which kind of provider:<br>OB/GYN<br>PCP<br>Midwife<br>Nurse<br>Other	Dichotomous (yes/no)
Current pregnancy provider sterilization counseling:	Have you discussed getting a tubal sterilization (also known as "getting your tubes tied" or a tubal ligation) with a healthcare provider during this pregnancy?	BL, T2, T3	Dichotomous (yes/no)	Dichotomous (yes/no)
Previous pregnancy provider sterilization counseling:	Have you discussed getting your tubes tied or having a tubal sterilization with your doctor or healthcare provider **before this pregnancy**?	BL	Dichotomous (yes/no)	Dichotomous (yes/no)
Age		BL	Continuous (month/year)	Continuous
Race/ethnicity		BL	Nominal<br><br>Hispanic or Latino (yes/no)<br>¡ Puerto Rican<br>¡ Cuban<br>¡ Mexican, Mexican American, or Chicana<br>¡ Central or South American<br>¡ Another Hispanic, Latina, or Spanish origin<br><br>Race:<br>¡ Black or African American<br>¡ White<br>¡ Asian<br>¡ Native Hawaiian or Pacific Islander<br>¡ American Indian or Native	Nominal:<br>Non-Hispanic White (ref)<br>Non-Hispanic Black<br>Hispanic/Latino<br>Other

			Alaskan	
Relationship status		BL	Nominal:<br>¡ Single<br>¡ Married<br>¡ Divorced/separated<br>¡ Not married but living with partner<br>¡ Widowed<br>¡ Other (*Specify*):	Nominal:<br>¡ Single<br>¡ Married<br>¡ Divorced/separated<br>¡ Not married but living with partner<br>¡ Widowed<br>¡ Other (*Specify*):
Gravida	"How many times have you been pregnant?"	BL	Count	Continuous
Parity	"How many times have you given birth"	BL	Count	Continuous
History of abortion		BL	Count	Dichotomous (yes/no)
Medical and mental health history	a) Hypertension/high blood pressure<br>b) History of lung clot<br>c) History of heart attack or stroke<br>d) Breast cancer<br>e) Liver tumor<br>f) HIV or AIDS<br>g) Diabetes<br>h) Migraine with aura<br>i) Lupus<br>j) Seizure disorder/epilepsy<br>k) Smoking<br>l) Major depression<br>m) Bipolar depression<br>n) Schizophrenia<br>o) Any other serious medical condition	BL	Dichotomous (yes/no)	Dichotomous (yes any medical/mental history; no-no history)
Any other serious medical condition		BL	Open-ended	Qualitative content analysis: code the open-responses into categories. If any categories are meaningful they can be added as a dichotomous baseline characteristic (because we didn't specifically survey on those conditions, though, we might have inaccurate data for people we assume to be a "No" when really they just didn't list it.
Pre-pregnancy weight (pounds)		BL	Continuous (pounds)	Continuous (pounds)
Height (feet and inches)		BL	Continuous (feet)<br>Continuous (inches)	Continuous (inches) using the formula:<br>Height= 12*(feet) + inches
CALCULATED: Body Mass Index ‡			BMI = kg/m2<br><br>Convert pounds to kilograms:<br><br>Kilos= Pounds/2.205<br><br>Convert feet/inches into meters:<br><br>Meters= [(feet x 12) + (inches)]/ 39.37	Continuous<br><br>Categorical:<br>1. BMI less than 18.5= underweight range.<br>2. 18.5 to <25= healthy weight range.<br>3. 25.0 to <30= overweight range.<br>4. 30.0 or higher= obesity range.
Gestational age	Are you currently less than 24 weeks pregnant?	BL		
Medicaid coverage	"Did Medicaid cover your pregnancy?	BL, T2, T3	Dichotomous (yes/no)	Dichotomous (yes=1, no=0)
Religion		BL	¡ No religion<br>¡ Protestant<br>¡ Catholic<br>¡ Other Christian	¡ No religion<br>¡ Protestant<br>¡ Catholic<br>¡ Other Christian

			¡ Jewish<br>¡ Muslim<br>¡ Other (*Specify*):	¡ Jewish<br>¡ Muslim<br>¡ Other (*Specify*):
Highest education		BL	¡ Less than a high school diploma<br>¡ High school diploma or GED<br>¡ Trade school or technical school<br>¡ Some college<br>¡ College degree<br>¡ Master's degree or higher	¡ Less than a high school diploma<br>¡ High school diploma or GED<br>¡ Trade school or technical school<br>¡ Some college<br>¡ College degree<br>¡ Master's degree or higher
Health literacy	How confident are you filling out medical forms by yourself?	BL	Ordinal:<br>¡ Not at all<br>¡ A little bit<br>¡ Somewhat<br>¡ Quite a bit<br>¡ Extremely	Ordinal:<br>¡ Not at all<br>¡ A little bit<br>¡ Somewhat<br>¡ Quite a bit<br>¡ Extremely
Annual income		BL	¡ 0 to $9,999<br>¡ $10,000 to $19,999<br>¡ $20,000 to $29,999<br>¡ $30,000 to $39,999<br>¡ $40,000 to $49,999<br>¡ $50,000 to $59,999<br>¡ $60,000 to $69,999<br>¡ $70,000 to $79,999<br>¡ $80,000 or more	3 categories:<br><br><100% FPL<br><200% FPL<br>200%+ FPL
People in household		BL	Count	
CALCULATED: Federal Poverty Level		BL	N/A	
Contraceptive method history:	Please indicate which, if any, methods of birth control you used right before this pregnancy. Say yes to all that apply.	BL	¡ Birth control pills<br>¡ Male Condom<br>¡ Partner's vasectomy<br>¡ Tubal sterilization<br>¡ Withdrawal or pulling out<br>¡ Contraceptive injection (Depo-Provera)<br>¡ Hormonal implant (Nexplanon)<br>¡ Natural family planning/rhythm method<br>¡ Female condom<br>¡ Sponge,diaphragm, or cervical cap<br>¡ Emergency contraception<br>¡ Spermicides<br>¡ Intrauterine device (IUD)<br>¡ Contraceptive patch<br>¡ Vaginal contraceptive ring/ Nuva Ring<br>¡ Other (*Specify*): ________________<br>¡ None<br>¡ Don't know or unsure	
In-person or virtual delivery of the Decision	*[noted by research assistant at UT site, where virtual and in-person recruitment is being used]*	BL	Dichotomous (in-person/virtual)	Dichotomous (in-person/virtual)

Aid				
Language of the decision aid	*[must be noted in the dataset manually; it is determined by which Decision Aid variables are completed by the participant--Spanish version pulls different variables from the Decision Aid]*	Within the Decision Aid	Dichotomous [English/Spanish]	Dichotomous [English/Spanish]

*Hahn TA, McKenzie F, Hoffman SM, Daggy J, Tucker Edmonds B. A prospective study on the effects of Medicaid regulation and other barriers to obtaining postpartum sterilization. *Journal of Midwifery & Women's Health*. 2018;64(2):186-193. doi:10.1111/jmwh.12909

†Consent for sterilization - hhs.gov. https://opa.hhs.gov/sites/default/files/2020-07/consent-for-sterilization-english-updated.pdf. Accessed August 11, 2022.

‡ Defining adult overweight & obesity. Centers for Disease Control and Prevention. https://www.cdc.gov/obesity/basics/adult-defining.html. Published June 3, 2022. Accessed August 11, 2022.

Statistical Analyses

Descriptive and Baseline Statistics

All primary and secondary outcomes will be described using sample means or proportions along with accompanying confidence intervals. Suitable transformations will be considered for continuous variables if there are substantial departures from normality. Baseline characteristics will be compared between the study arms using two-sample t-tests or chi-square tests, as appropriate (see Outcome Variables tables above).

Primary and Secondary Hypotheses

For the following primary and secondary hypotheses, we will conduct an intention-to-treat analysis using linear or logistic regression to test intervention effects controlling for study site. The beta coefficient and corresponding 97.5% confidence interval will be used to assess the effect of the intervention.

- **Primary Hypothesis a:** Compared to women who receive usual care alone, women randomized to the decision aid arm will have greater knowledge about sterilization and alternative options at T1

- **Primary Hypothesis b:** Compared to women who receive usual care alone, women randomized to the decision aid arm will have lower decisional conflict in postpartum contraceptive choice at T1

- **Secondary Hypothesis a:** Compared to women who receive usual care alone, women randomized to the decision aid arm will also be less likely to select sterilization and more likely to select an alternative method at T1

- **Secondary Hypothesis b:** Compared to women who receive usual care alone, women randomized to the decision aid arm will also be more satisfied with their decision at 3-month follow-up (T3)

Handling Missing Data

We will record reasons for dropout at T2 and T3 and investigate any relationship with study arm. We will also compare baseline characteristics between women who do and do not complete the study. With regard to missing data, we will investigate the mechanism and degree of missingness using established techniques. If the amount of missingness is great, we will compare various imputation techniques to determine how robust the overall inferences are.[38]

Exploratory Hypotheses

For the following exploratory hypotheses, regression will be used to test intervention effects controlling for study site. The beta coefficient and corresponding 97.5% confidence interval will be used to assess the effect of the intervention.

- **Pre- and Post-*Dobbs* SCOTUS Decision Exploratory Analyses:** regression analyses are adjusted for study site **note state interaction effects below**
  - Date for the *Dobbs* decision can be categorized in 3 ways:
    - Dichotomized as before June 24, 2022 and June 24, 2022 or later (the verdict)
    - Dichotomized as before May 1, 2022 and May 1, 2022 or later (the leak)
    - Categorical: before May 1; May 1- June 23; June 23 or later (would measure effect of the leaked news and then effect of the full impact)
  - How did the *Dobbs* SCOTUS decision impact recruitment?
    - Recruitment changes pre- and post-Dobbs
      - More interest in joining study
      - Differences in recruitment across gravida and parity
  - How has it affected our outcomes of interest?
    - The desire for sterilization at baseline
    - Decisional conflict about contraceptive method selection at T1 predicted by date
    - Ease in contraceptive decision making at T1 and T2 predicted by date
    - Certainty about sterilization decision at T1 and T2 predicted by date
    - Contraceptive method selected at T1 and T2 predicted by date
    - Contraceptive method used at T3 predicted by date

        - **Interaction effect between date and state on all outcomes (for every outcome)**

- **<u>Additional Sensitivity Analyses for Primary and Secondary Outcomes</u>**
    - We will run additional sensitivity analyses to test robustness of the intervention effects after adjusting for any significant differences in baseline characteristics (to be identified during analysis) between the intervention and control group that are also associated with the outcomes of interest:
        - 1) sterilization knowledge at T1
        - 2) decisional conflict at T1
        - 3) contraceptive method selected at T1
        - 4) satisfaction with decision-making at T3
    - We will run additional sensitivity analyses to test robustness of the intervention effects after adjusting for differences in:
        - T3 Outcomes (satisfaction with decision-making at T3):
            - Provider sterilization counseling between T1 and T3 (composite variable of this question at T3 about counseling during this pregnancy isolating those who said they already received this counseling prior to or during this pregnancy at baseline)
            - Provider contraceptive counseling between T1 and T3
            - Medicaid consent signed between T1 and T3
            - Provider sterilization and/or Medicaid consent between T1 and T3

- **<u>Subgroup Analyses (Interactions, no *a priori* hypothesis, not powered) of the Primary Outcomes by:</u>**
  Regression model needs to control for site, including direct effects of the decision aid and the variable of interest below. Check for collinearity (VIF with a cut-off of 10)
    - Race/ethnicity (non-Hispanic Black, non-Hispanic White, Hispanic, non-Hispanic Other)
        - **<u>Knowledge=decision aid + site + race/ethnciity + DA#Race/ethnicity</u>**
        - **<u>Decisional conflict= decision aid + site + race/ethnciity + DA#Race/ethnicity</u>**

    - Age (categorized for this analysis: <25, 25+)
        - **<u>Knowledge= decision aid + site + age + DA#age</u>**

    - Completed Decision Aid in-person or virtual
    - Recruitment Site (California, Pennsylvania, Tennessee)
    - Language of the consent form (English vs Spanish)
    - Education level (HS or less including GED vs. any post-high school or more)
    - Provider counseling/consent form signed at T1
        - Provider sterilization counseling at baseline (before this pregnancy, during this pregnancy)

- **<u>Participant Satisfaction with Current Contraceptive Method(s) at Time 3:</u>** Compared to women who receive usual care alone, women randomized to the decision aid arm will report greater satisfaction with their current contraceptive method(s) at Time 3

- **<u>Desire for sterilization:</u>** variable will be used to assess baseline differences (pre-decision aid for intervention group, T1 for control), changes from baseline to T1 for intervention groups, and differences between post-decision aid for intervention group vs T1 for control group.
    - To test exploratory hypotheses, we will first use linear regression (using the continuous measure 1-5 where higher score indicates higher desire for sterilization) to test intervention effects (pre-intervention baseline vs post-intervention T1; post-intervention vs T1 control), controlling for study site.
    - We will also use ordinal regression (using the recoded 3, 4, and 5 values where higher score indicates higher strength of desire but not for/against sterilization) to test intervention effects controlling for study site.

- **<u>Participant Knowledge about Tubal Sterilization at Time 2:</u>** Compared to women who receive usual care alone, women randomized to the decision aid arm will have greater knowledge about sterilization and alternative options at T2.

- *Sensitivity Analyses*: We will run sensitivity analyses to test robustness of the intervention effects after adjusting for differences in:
  - Provider sterilization counseling between T1 and T2 ("since our last meeting")
  - Provider contraceptive counseling between T1 and T2 ("since our last meeting")
  - Medicaid consent signed between T1 and T2 ("since our last meeting")
  - Provider sterilization and/or Medicaid consent signed between T1 and T2 ("since our last meeting")

- **Participant Decisional Conflict in Postpartum Contraceptive Choice at Time 2:** Compared to women who receive usual care alone, women randomized to the decision aid arm will have lower decisional conflict in postpartum contraceptive choice at Time 2
- *Sensitivity Analyses*: We will run sensitivity analyses to test robustness of the intervention effects after adjusting for differences in:
  - Provider sterilization counseling between T1 and T2 ("since our last meeting")
  - Provider contraceptive counseling between T1 and T2 ("since our last meeting")
  - Medicaid consent signed between T1 and T2 ("since our last meeting")
  - Provider sterilization and/or Medicaid consent signed between T1 and T2 ("since our last meeting")

- **Contraceptive Method Selected at Time 2:** Compared to women who receive usual care alone, women randomized to the decision aid arm will be less likely to select sterilization at T2 and more likely to select an alternative method

- **Contraceptive Method(s) Used at Time 3:** Compared to women who receive usual care alone, women randomized to the decision aid arm will be less likely to select sterilization at T3 and more likely to select an alternative method

- **Ease in Contraceptive Decision Making at Time 1:** Compared to women who receive usual care alone, women randomized to the decision aid arm will report greater ease in contraceptive decision making at Time 1

- **Ease in Contraceptive Decision Making at Time 2:** Compared to women who receive usual care alone, women randomized to the decision aid arm will report greater ease in contraceptive decision making at Time 2

- **Certainty about Tubal Sterilization Decision at Time 1:** Compared to women who receive usual care alone, women randomized to the decision aid arm will report a) greater certainty about tubal sterilization decision at Time 1 and b) greater certainty in general, regardless of whether that's for or against tubal sterilization
  - Multinomial regression (certain about not getting it vs. uncertain vs. certain about getting it) will be used to test intervention effects controlling for study site
  - Linear regression will be used (5-10, uncertain to certain) to test intervention effects controlling for study site
  - Logistic regression will be used (uncertain vs certain) to test intervention effects controlling for study site

- **Certainty about Tubal Sterilization Decision at Time 2:** Compared to women who receive usual care alone, women randomized to the decision aid arm will report a) greater certainty about tubal sterilization decision at Time 2 and b) greater certainty in general, regardless of whether that's for or against tubal sterilization
  - Multinomial regression (certain about not getting it vs. uncertain vs. certain about getting it) will be used to test intervention effects controlling for study site
  - Linear regression will be used (5-10, uncertain to certain) to test intervention effects controlling for study site
  - Logistic regression will be used (uncertain vs certain) to test intervention effects controlling for study site

- **Participant Confidence in Contraceptive Method Selection at Time 3:** Compared to women who receive usual care alone, women randomized to the decision aid arm will report greater confidence that their current contraceptive method(s) is "right for them" at Time 3

- **Subsequent Pregnancies Since Delivery at Time 3:** Compared to women who receive usual care alone, women randomized to the decision aid arm will report lower likelihood of having a subsequent pregnancy at Time 3

Exploratory Analyses in Specific Subset(s) of the Sample

**Note: these analyses on specific subsets of the sample no longer rely on the randomization process. Each of the analyses below will need to assess for potential confounding factors and control for those factors in addition to study site:*

1) Among People who Wanted Sterilization at Time 2:

- What Contraceptive Method(s) are Used at Time 3: binary logistic regression will be used to test intervention effects on odds of sterilization vs all other methods controlling for study site and other potential confounders
    - We specifically hypothesize that intervention group members will be less likely to report using sterilization as compared to the control group

2) Among People who Wanted Sterilization at Time 2 but Did Not have Sterilization at T3:

- What are their Reasons for Contraceptive Method(s) Mismatch: a series of logistic regressions will be used to test intervention effects on *reasons for mismatch* controlling for study site and potential confounders
    - We specifically hypothesize that intervention group members will be less likely to report logistical barriers.
        - Logistical barriers are defined as any of the following reasons: Financial concerns, Method was unavailable/too difficult to obtain, Method was too difficult to use

3) Among people who had sterilization counseling:

- What is Participant Satisfaction with Sterilization Counseling at Time 3: linear regression to test intervention effects controlling for study site and potential confounders
    - Compared to women who receive usual care alone, women randomized to the decision aid arm will report greater satisfaction with sterilization counseling at Time 3

Figure 1. CONSORT Diagram

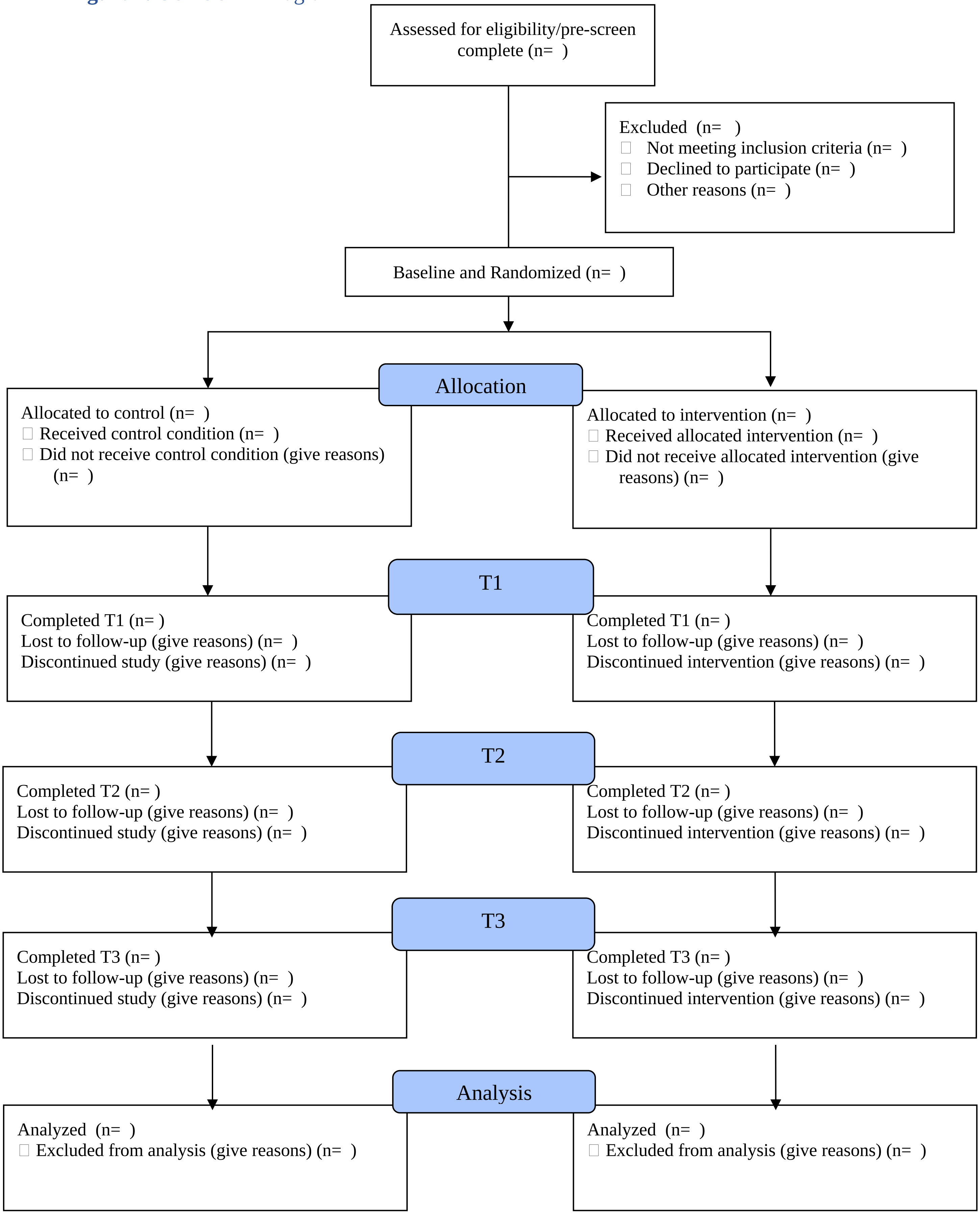

References

1. National Survey of Family Growth. Cycle 2017-2019. Published 2021. Accessed January 1, 2021. http://www.cdc.gov/nchs/nsfg.htm

2. Borrero S, Abebe K, Dehlendorf C, et al. Racial variation in tubal sterilization rates: role of patient-level factors. *Fertil Steril*. 2011;95(1):17-22. doi:10.1016/j.fertnstert.2010.05.031

3. Shih G, Dubé K, Sheinbein M, Borrero S, Dehlendorf C. He's a Real Man: A Qualitative Study of the Social Context of Couples' Vasectomy Decisions Among a Racially Diverse Population. *Am J Mens Health*. 2013;7(3):206-213. doi:10.1177/1557988312465888

4. White K, Hopkins K, Potter JE, Grossman D. Knowledge and Attitudes about Long-Acting Reversible Contraception Among Latina Women Who Desire Sterilization. *Women's Health Issues*. 2013;23(4):e257-e263. doi:10.1016/j.whi.2013.05.001

5. Zite N, Wallace LS. Use of a Low-Literacy Informed Consent Form to Improve Women's Understanding of Tubal Sterilization: A Randomized Controlled Trial. *Obstet Gynecol*. 2011;117(5):1160-1166. doi:10.1097/AOG.0b013e318213cbb1

6. Borrero S, Nikolajski C, Rodriguez KL, Creinin MD, Arnold RM, Ibrahim SA. "Everything I Know I Learned from My Mother...or Not": Perspectives of African-American and White Women on Decisions About Tubal Sterilization. *J Gen Intern Med*. 2009;24(3):312-319. doi:10.1007/s11606-008-0887-3

7. Henshaw SK, Singh S. Sterilization Regret Among U.S. Couples. *Family Planning Perspectives*. 1986;18(5):238. doi:10.2307/2134990

8. Schmidt JE, Hillis SD, Marchbanks PA, Jeng G, Peterson HB. Requesting information about and obtaining reversal after tubal sterilization: findings from the U.S. collaborative review of sterilization. *Fertility and Sterility*. 2000;74(5):892-898. doi:10.1016/S0015-0282(00)01558-2

9. Borrero S, Zite N, Potter JE, Trussell J, Smith K. Potential unintended pregnancies averted and cost savings associated with a revised Medicaid sterilization policy. *Contraception*. 2013;88(6):691-696. doi:10.1016/j.contraception.2013.08.004

10. Gilliam M, Davis SD, Berlin A, Zite N. A qualitative study of barriers to postpartum sterilization and women's attitudes toward unfulfilled sterilization requests. *Contraception*. 2008;77(1):44-49. doi:10.1016/j.contraception.2007.09.011

11. Potter JE, White K, Hopkins K, et al. Frustrated Demand for Sterilization Among Low-Income Latinas in El Paso, Texas. *Perspect Sex Repro H*. 2012;44(4):228-235. doi:10.1363/4422812

12. Thurman A, Janecek T. One-year follow-up of women with unfulfilled postpartum sterilization requests. *Obstet Gynecol*. 2010;116(5):1071-1077.

13. Thurman A, Harvey D, Shain R. Unfulfilled postpartum sterilization requests. *The Journal of Reproductive Medicine*. 2009;54(8):567-472.

14. Zite N, Wuellner S, Gilliam M. Barriers to obtaining a desired postpartum tubal sterilization. *Contraception*. 2006;73(4):404-407. doi:10.1016/j.contraception.2005.10.014

15. Zite N, Wuellner S, Gilliam M. Failure to Obtain Desired Postpartum Sterilization: Risk and Predictors: *Obstet Gynecol*. 2005;105(4):794-799. doi:10.1097/01.AOG.0000157208.37923.17

16. Brown S, Eisenberg L. *The Best Intentions: Unintended Pregnancy and the Well-Being of Children and Families*. National Academy Press; 1995.

17. Gipson J, Koenig M, Hindin M. The effects of unintended pregnancy on infant, child, and parental health: a review of the literature. *Studies in Family Planning*. 2008;39(1):18-38.

18. Institute of Medicine. *Clinical Preventative Services for Women Closing the Gaps*. The National Academies Press; 2011.

19. Najman JM, Morrison J, Williams G, Andersen M, Keeping JD. The mental health of women 6 months after they give birth to an unwanted baby: A longitudinal study. *Social Science & Medicine*. 1991;32(3):241-247. doi:10.1016/0277-9536(91)90100-Q

20. Department of Health, Education, and Welfare. *Sterilization Under Medicaid*. Vol 42.; 1978.

21. Stern AM. Sterilized in the name of public health: race, immigration, and reproductive control in modern California. *Am J Public Health*. 2005;95(7):1128-1138. doi:https://doi.org/10.2105/AJPH.2004.041608

22. Shapiro TM, Fisher W, Diana A. Family planning and female sterilization in the United States. *Soc Sci Med*. 1983;17(23):1847-1855. doi:10.1016/0277-9536(83)90161-2

23. Zite N, Philipson SJ, Wallace LS. Consent to Sterilization section of the Medicaid-Title XIX form: is it understandable? *Contraception*. 2007;75(4):256-260. doi:10.1016/j.contraception.2006.12.015

24. Harris LH, Wolfe T. Stratified reproduction, family planning care and the double edge of history. *Current Opinions in Obstetrics and Gynecology*. 2014;26(6):539-544. doi:10.1097/GCO.0000000000000121.

25. Brown BP, Chor J. Adding Injury to Injury: Ethical Implications of the Medicaid Sterilization Consent Regulations. *Obstetrics & Gynecology*. 2014;123(6):1348-1351. doi:10.1097/AOG.0000000000000265

26. Access to postpartum sterilization. Committee Opinion No. 530. American College of Obstetricians and Gynecologists. *Obstet Gynecol*. 2012;120(1):212-215.

27. Borrero S, Zite N, Creinin MD. Federally Funded Sterilization: Time to Rethink Policy? *Am J Public Health*. 2012;102(10):1822-1825. doi:10.2105/AJPH.2012.300850

28. Borrero S, Zite N, Potter JE, Trussell J. Medicaid Policy on Sterilization — Anachronistic or Still Relevant? *N Engl J Med*. 2014;370(2):102-104. doi:10.1056/NEJMp1313325

29. Henderson CE, Ringel LE, Nezan H, Rezai S, Sherman S. Postpartum Sterilization: Underserved Women Struggle with Bureaucratic Laws and Regulations. *NYSBA Health Law Journal*. 2014;19(2):49-53.

30. Moaddab A, McCullough LB, Chervenak FA, et al. Health care justice and its implications for current policy of a mandatory waiting period for elective tubal sterilization. *Am J Obstet Gynecol*. 2015;212(6):736-739. doi:10.1016/j.ajog.2015.03.049

31. Darney PD. New kinds of injustice for women? *Am J Obstet Gynecol*. 2015;212(6):693-694. doi:10.1016/j.ajog.2015.04.021

32. Block-Abraham D, Arora K, Tate D, Gee R. Medicaid Consent to Sterilization forms: historical, practical, ethical, and advocacy considerations. *Clin Obstet Gynecol*. 2015;58(2):409-417. doi:10.1097/GRF.0000000000000110

33. Johnson CG. Female inmates sterilized in California prisons without approval. *RevealNews*. https://revealnews.org/article/female-inmates-sterilized-in-california-prisons-without-approval/. Published July 7, 2013. Accessed February 23, 2022.

34. Evans ML, Qasba N, Shah Arora K. COVID-19 highlights the policy barriers and complexities of postpartum sterilization. *Contraception*. 2021;103(1):3-5. doi:10.1016/j.contraception.2020.10.006

35. Sun Q. *Predicting Downstream Effects of High Decisional Conflict: Meta-Analyses of the Decisional Conflict Scale*. University of Ottawa; 2005. Accessed February 23, 2022. https://ruor.uottawa.ca/handle/10393/27050

36. Brehaut JC, O'Connor AM, Wood TJ, et al. Validation of a Decision Regret Scale. *Med Decis Making*. 2003;23(4):281-292. doi:10.1177/0272989X03256005

37. Cohen J. *Statistical Power Analysis for the Behavioral Sciences*. 2nd ed. Routledge; 1988.

38. Little J, Rubin D. *Statistical Analysis with Missing Data*. 2nd ed. Wiley; 2002.
