# Supplementary material for: A Decision Aid to Support Tubal Sterilization Decision-Making Among Pregnant Women: The MyDecision/MiDecisión Randomized Clinical Trial
Source: JAMA Netw Open. 2024 Mar 19;7(3):e242215. doi: 10.1001/jamanetworkopen.2024.2215 (PMC10951734; doi:10.1001/jamanetworkopen.2024.2215)
Supplement: Supplement 2. — eTable 1. Participant Demographics and Baseline Characteristics eTable 2. Evaluation of the Effect of MyDecision/MiDecisión on the Primary Outcomes, by Planned Subgroups [file jamanetwopen-e242215-s002.pdf]

Supplementary Online Content

Borrero S, Mosley EA, Wu M, et al. A decision aid to support tubal sterilization decision-making among pregnant women: the MyDecision/MiDecisión randomized clinical trial. *JAMA Netw Open*. 2024;7(3):e242215. doi:10.1001/jamanetworkopen.2024.2215

eTable 1. Participant Demographics and Baseline Characteristics

eTable 2. Evaluation of the Effect of MyDecision/MiDecisión on the Primary Outcomes, by Planned Subgroups

This supplementary material has been provided by the authors to give readers additional information about their work.

© 2024 Borrero S et al. *JAMA Network Open*.

eTable 1. Participant demographics and baseline characteristics (n=350)

Variable	All Participants (n=350)[1]	San Francisco (n=75)	Pittsburgh (n=116)	Tennessee (n=159)
	Mean/% (SD/n)	Mean/% (SD/n)	Mean/% (SD/n)	Mean/% (SD/n)
Age in years	29.7 (5.1)	31.9 (5.3)	29.7 (5.0)	28.8 (4.7)
Self-identified race/ethnicity				
Hispanic or Latino	26.0% (91)	89.3% (67)	3.4% (4)	12.6% (20)
Non-Hispanic white	39.1% (137)	4% (3)	26.7% (31)	64.8% (103)
Non-Hispanic Black	26.0% (91)	1.3% (1)	59.5% (69)	13.2% (21)
Non-Hispanic multiracial	4.0% (14)	1.3% (1)	5.2% (6)	4.4% (7)
Non- Hispanic other	4.0% (14)	2.7% (2)	4.3% (5)	4.4% (7)
Education				
< HS	11.7% (41)	20% (15)	7.8% (9)	10.7% (17)
HS or GED	47.1% (165)	46.7% (35)	44.8% (52)	49.1% (78)
Some college or more	40.9% (143)	33.3% (25)	46.6% (54)	40.3% (64)
Relationship status				
Single or never married	27.1% (95)	28% (21)	23.3% (27)	17% (27)
Married or cohabiting	64.0% (224)	61.3% (46)	81.0% (94)	74.8% (119)
Previously married	3.7% (13)	5.3% (4)	1.7% (2)	4.4% (7)
Other or Missing	5.1% (18)	5.3% (4)	6.9% (8)	3.8% (6)
Federal poverty level[2]				
<100%	56.3% (197)	65.3% (49)	56.9% (66)	51.6% (82)
100-199%	38.3% (134)	30.7% (23)	33.6% (39)	45.3% (72)
>200%	2.9% (10)	2.7% (2)	2.6% (3)	3.1% (5)
Missing	2.6% (9)	1.3% (1)	6.9% (8)	0% (0)
Poor/marginal health literacy	16.9% (59)	33.3% (25)	12.1% (14)	12.6% (20)
Number of children (excluding current pregnancy)	2.16 (1.4)	1.9 (1.4)	2.3 (1.6)	2.2 (1.3)
Gestational age	16.1 (12.7-20.1)	15.1 (12.7-17.4)	16.9 (12.8-21.4)	15.9 (10.9-19.7)
Reported receiving any contraceptive counseling during this pregnancy	52.9% (185)	34.7% (26)	41.4% (48)	69.6% (111)
Reported sterilization counseling during this pregnancy	46.3% (162)	24.0% (18)	34.5% (40)	65.4% (104)
Reported signing the Medicaid consent form	2.3% (8)	0% (0)	0% (0)	5% (8)

[1] Response rates varied across site due to differences in recruitment approaches. Overall, 350 participants completed T1 assessments out of 549 pre-screened (63.8%). Response rates by site were: 75 out of 112 pre-screened (67.0%) in San Francisco; 116 out of 257 pre-screened (45.1%) in Pittsburgh; 159 out of 180 (88.3%) in Knoxville.

[2] Defined by patients responding to a 5-point single-item literacy screening question, "How confident are you filling out medical forms by yourself?" with "somewhat confident," "a little bit confident," or "not at all confident."

© 2024 Borrero S et al. *JAMA Network Open*.

eTable 2: Evaluation of the effect of MyDecision/MiDecisión on the primary outcomes, by planned subgroups

Subgroup	Knowledge about tubal sterilization Mean difference (95% CI)	Decisional conflict Mean difference (95% CI)
Self-identified race/ethnicity (*overall interaction p-value*)	*0.180*	*0.456*
Non-Hispanic White (n=137)	15.4 (9.5, 21.4)	-4.0 (-8.8, 0.8)
Non-Hispanic Black (n=91)	23.4 (14.7, 32.1)	-5.1 (-11.7, 1.6)
Hispanic (n=91)	25.5 (18.1, 33.0)	-10.7 (-20.2, -1.2)
Non-Hispanic Other (n=28)	25.8 (8.8, 42.8)	-1.9 (-15.6, 11.9)
Language (*overall interaction p-value*)	*0.205*	*0.003*
English (n=294)	19.7 (15.3, 24.1)	-3.7 (-7.0, -0.3)
Spanish (n=56)	26.8 (17.1, 36.5)	-18.2 (-31.6, -4.9)
Age (*overall interaction p-value*)	*0.217*	*0.810*
<30 years (n=187)	18.3 (13.0, 23.7)	-4.7 (-9.7, 0.3)
30 years+ (n=163)	23.5 (17.3, 29.7)	-6.3 (-11.9, -0.8)
Mode of delivery (*overall interaction p-value*)	*0.957*	*0.532*
In-person (n=37)	20.5 (10.7, 30.3)	-2.4 (-13.0, 8.2)
Virtual (n=312)	20.9 (16.5, 25.2)	-6.3 (-10.2, -2.3)
Study site (*overall interaction p-value*)	*0.087*	*0.186*
Pittsburgh (n=116)	17.1 (9.2, 25.1)	-3.8 (-8.8, 1.2)
Knoxville (=159)	19.5 (14.1, 24.9)	-4.1 (-9.0, 0.8)
San Francisco (n=75)	29.2 (20.8, 37.7)	-12.3 (-23.7, -0.9)
Education (*overall interaction p-value*)	*0.154*	*0.826*
High school or less (n=206)	23.2 (18.1, 28.3)	-6.2 (-10.8, -1.5)
More than high school (n=143)	17.2 (10.9, 23.6)	-5.4 (-11.4, 0.7)
Reported receiving sterilization counseling during this pregnancy (*overall interaction p-value*)	*0.119*	*0.557*
Yes (n=162)	17.4 (11.5, 23.4)	-4.4 (-9.5, 0.7)
No (n=188)	23.8 (18.3, 29.3)	-6.5 (-11.7, -1.3)

Note: Mean differences and confidence intervals and the interaction effects and their p-values were calculated using multivariate linear regression adjusted for recruitment site; virtual appointments comprise both those conducted entirely virtually, and those described as a combination of in-person and virtual

© 2024 Borrero S et al. *JAMA Network Open*.
