# Supplementary material for: A Decision Aid to Support Tubal Sterilization Decision-Making Among Pregnant Women: The MyDecision/MiDecisión Randomized Clinical Trial
Source: JAMA Netw Open. 2024 Mar 19;7(3):e242215. doi: 10.1001/jamanetworkopen.2024.2215 (PMC10951734; doi:10.1001/jamanetworkopen.2024.2215)
Supplement: Supplement 3. — Data Sharing Statement [file jamanetwopen-e242215-s003.pdf]

Data Sharing Statement

Borrero. A Decision Aid to Support Tubal Sterilization Decision-Making Among Pregnant Women. *JAMA Netw Open*. Published March 19, 2024.
doi:10.1001/jamanetworkopen.2024.2215

Data
Data available: No

Additional Information
Explanation for why data not available: We would make decisions about whether to offer de-identified data and/or data dictionary to others on a case-by-case basis.
